# Comparison of Resampling Algorithms to Address Class Imbalance when Developing Machine Learning Models to Predict Foodborne Pathogen Presence in Agricultural Water

**DOI:** 10.3389/fenvs.2021.701288

**Published:** 2021-06-29

**Authors:** Daniel Lowell Weller, Tanzy M. T. Love, Martin Wiedmann

**Affiliations:** 1 Department of Biostatistics and Computational Biology, University of Rochester, Rochester, NY, United States; 2 Department of Environmental and Forest Biology, State University of New York, Environmental Science and Forestry, Syracuse, NY, United States; 3 Department of Food Science, Cornell University, Ithaca, NY, United States

**Keywords:** Listeria, Listeria (L.) monocytogenes, machine learning, predictive modeling, agricultural water, food safety, class imbalance, SMOTE (synthetic minority over-sampling technique)

## Abstract

Recent studies have shown that predictive models can supplement or provide alternatives to *E. coli*-testing for assessing the potential presence of food safety hazards in water used for produce production. However, these studies used balanced training data and focused on enteric pathogens. As such, research is needed to determine 1) if predictive models can be used to assess *Listeria* contamination of agricultural water, and 2) how resampling (to deal with imbalanced data) affects performance of these models. To address these knowledge gaps, this study developed models that predict nonpathogenic *Listeria* spp. (excluding *L. monocytogenes*) and *L. monocytogenes* presence in agricultural water using various combinations of learner (e.g., random forest, regression), feature type, and resampling method (none, oversampling, SMOTE). Four feature types were used in model training: microbial, physicochemical, spatial, and weather. “Full models” were trained using all four feature types, while “nested models” used between one and three types. In total, 45 full (15 learners*3 resampling approaches) and 108 nested (5 learners*9 feature sets*3 resampling approaches) models were trained per outcome. Model performance was compared against baseline models where *E. coli* concentration was the sole predictor. Overall, the machine learning models outperformed the baseline *E. coli* models, with random forests outperforming models built using other learners (e.g., rule-based learners). Resampling produced more accurate models than not resampling, with SMOTE models outperforming, on average, oversampling models. Regardless of resampling method, spatial and physicochemical water quality features drove accurate predictions for the nonpathogenic *Listeria* spp. and *L. monocytogenes* models, respectively. Overall, these findings 1) illustrate the need for alternatives to existing *E. coli*-based monitoring programs for assessing agricultural water for the presence of potential food safety hazards, and 2) suggest that predictive models may be one such alternative. Moreover, these findings provide a conceptual framework for how such models can be developed in the future with the ultimate aim of developing models that can be integrated into on-farm risk management programs. For example, future studies should consider using random forest learners, SMOTE resampling, and spatial features to develop models to predict the presence of foodborne pathogens, such as *L. monocytogenes*, in agricultural water when the training data is imbalanced.

## INTRODUCTION

Given the number of high-profile, multistate outbreaks linked to fresh produce over the last two decades, preharvest produce safety is of increasing concern to government and industry stakeholders as well as consumers (Newell et al., 2010; Zhu et al., 2017). This is evidenced by the publication or revision of national regulations [e.g., the United States Food Safety Modernization Act (FSMA); Kleinwechter and Grethe (2006), FDA (2015), UN FAO (2017)], inter-governmental agreements [e.g., Faour-Klingbeil and Todd (2018), US FDA (2020)], voluntary grower agreements [e.g., the Leafy Greens Marketing Agreement; California Leafy Greens Marketing Agreement (2017)], and food safety guidance documents [e.g., Chapin et al. (2020), Corona et al. (2010), Gorny (2005), McEntire et al. (2019), National Berry Crops Initiative (2009), Nutrition, n.d.; Osborne et al. (2020), US FDA (2017)] as well as stakeholder feedback in surveys and at topical summits (Minor et al., 2019; Wall et al., 2019). Despite the widespread recognition that produce safety is a concern, there is some debate over the best way to manage preharvest environments to mitigate contamination risks (Wall et al., 2019). For instance, FSMA’s Produce Safety rule established a microbial water quality standard for surface water used for produce production (FDA, 2015). The standard states that growers must create a microbial water quality profile for each water source using 20 water samples collected over 2–4 years, and that the geometric mean and 90^th^ percentile of *E. coli* in these samples must be <126 CFU/100-ml and <410 CFU/100-ml, respectively (FDA, 2015). However, recent studies conducted in several US produce-growing regions (e.g., Southeast, Southwest, Northeast) have found that compliance with the proposed standard is not associated with a reduced risk of pathogen presence at the time of water use (Havelaar et al., 2017; Truitt et al., 2018; Weller et al., 2020b). Indeed, Havelaar et al. (2017) used data from Florida ponds to examine the predictive accuracy of the proposed standard and found that 1) variability in log10 *E. coli* levels was three-fold higher than the estimates used when drafting the standard, and 2) the sample size in the proposed standard (N = 20 samples) failed to capture this variability resulting in imprecise mean and 90^th^ percentiles estimates. Havelaar et al. (2017) also noted that this bias was exacerbated by limitations associated with *E. coli* enumeration methods. In addition, to specific concerns about the proposed FSMA standard, there is also considerable debate in the scientific literature, and produce safety community, about the efficacy of using *E. coli* to indicate the potential presence of food safety hazards in agricultural water. Indeed, multiple studies failed to find an association between *E. coli* levels and foodborne pathogen detection, found a negative association between *E. coli* levels and pathogen detection, or found that the direction and strength of this association was region, waterway, and/or pathogen-specific [e.g., (Harwood et al., 2005; McEgan et al., 2013; Bradshaw et al., 2016; Weller et al., 2020b)]. Thus, there is a clear need for alternative strategies for identifying produce safety hazards in surface waterways that provide water for produce production.

Interest in the application of predictive modeling, machine learning, and other computational approaches to food production has also increased over the past few decades. Due to the emergence of digital agriculture and the concomitant recognition of preharvest produce safety as a public health concern, there has been substantial interest in the use of digital agriculture for preharvest produce safety applications. Despite this interest, only a limited number of studies have developed and tested machine learning models to predict when and where foodborne pathogens are likely to be present in produce pre-harvest environments. Specifically, the authors are aware of 1) a New York study that developed and validated models to predict *Listeria* spp., and *L. monocytogenes* presence in produce field soils (Strawn et al., 2013; Weller et al., 2016), 2) a Florida study that developed and validated models to predict *Salmonella* presence in irrigation ponds (Polat et al., 2019), and 3) a New York study that developed and validated models to predict *Salmonella* and pathogenic *E. coli* presence in streams used for irrigation (Weller et al., 2020c). Overall, the results from these studies are encouraging; each study used an independent dataset for model validation, and found that predictive models were able to accurately predict pathogen presence and/or outperform baseline learners (Weller et al., 2016; Polat et al., 2019; Weller et al., 2020c). Moreover, studies that developed models to predict microbial contamination of other environments, such as recreational water and poultry farm soils, also concluded that machine learning models could be a useful tool for managing microbial hazards in the given environment (Efstratiou et al., 2009; Francy et al., 2013; Francy et al., 2014; Golden et al., 2019). However, almost all of these studies used a continuous outcome (e.g., concentration of the bacteria), used balanced training data or did not account for imbalance in the training data. Imbalanced data refers to when there are substantially fewer positive samples than negatives samples (or vice versa). This causes a problem since predictive models trained using imbalanced data can achieve relatively good accuracy by assigning the majority class to all samples (e.g., predicting all samples to be negative). Thus, there is limited evidence from applied produce safety studies on how different strategies for addressing imbalance can affect model performance. Similarly, no peer-reviewed study, to the author’s knowledge, has developed models to predict *Listeria* contamination of surface water used for produce production. Thus, the primary aims of this study were to determine 1) if machine learning could be used to develop models that accurately predict *Listeria* presence in agricultural water sources in the Northeastern US, and 2) the impact on predictive performance of different methods for dealing with imbalanced training data. Since collecting certain data types [e.g., field-collected, lab-generated microbial water quality data vs. weather data publicly available through an online portal] requiredifferent levels of capital, computational, and training investment to growers, a secondary aim of this study was to assess the relative information gain associated with using different feature types to build predictive models. The models developed here are not deployable models, and should not be used to guide on-farm decision-making. Instead, this study provides the conceptual framework that future studies can build upon to develop and incorporate field-ready models into on-farm decision-support tools (i.e., to develop deployable models). As such, this study was designed to complement existing studies that provide guidance on how machine learning approaches can be used to develop models to predict enteric pathogen presence in agricultural water using balanced training data Polat et al. (2019), Weller et al. (2020c), and *Listeria* spp. presence in field environments (Weller et al., 2016; Golden et al., 2019). While we acknowledge that *Salmonella* and pathogenic *E. coli* are the primary organisms of concern in surface water used for produce production, *Listeria* spp. and *L. monocytogenes* were used as models organisms here because 1) we lacked access to suitable (i.e., imbalanced) data on *Salmonella* and pathogenic *E. coli* contamination of agricultural waterways, and 2) *L. monocytogenes* is a foodborne pathogen of concern whose presence in agricultural water could lead to recalls and illness when contamination carries through to the finished product (Garner and Kathariou, 2016).

## MATERIALS AND METHODS

### Study design

This study used the datasets collected in 2017 Weller et al. (2020b) and 2018 Weller et al. (2020a) to test and train the models, respectively. While these data were previously published, the published studies focused on 1) characterizing associations between pathogen detection and environmental factors, 2) identifying sources of pathogen contamination, and 3) assessing the impact of sampling and laboratory methods on pathogen detection (Weller et al., 2020a; Weller et al., 2020b). Conversely, the current study focuses on the 1) development and comparison of predictive models using different algorithms and feature types, 2) impact of resampling methods (to address data imbalance) on model performance, and 3) identification of features that drive model accuracy. Moreover, unlike previous, applied studies that developed models to predict the presence of food safety hazards in agricultural water using balanced presence-absence Polat et al. (2019), Weller et al. (2020c) or continuous Weller et al. (2021) data, the current study focuses on predicting *Listeria* contamination using moderately (nonpathogenic *Listeria* spp.) and severely (*L. monocytogenes*) imbalanced presence-absence data (Table 1).

The sampling and laboratory methods for the training and test data were the same except for differences in the 1) number of sampling sites (6 in 2017, and 68 in 2018), and 2) frequency of sampling (15–34 visits per site in 2017, and 2 to 3 visits per site in 2018; Table 1). The sampled streams were located in the same geographic region (Upstate New York), and each dataset represents a single growing season (May to August in 2017, and April to October in 2018). At each sampling visit, 1) a 10-L grab sample (GS) was collected and tested for *Listeria* spp. and *L. monocytogenes,* 2) a 1-L GS was collected for *E. coli* enumeration, and 3) physicochemical water quality data were collected as previously described (Weller et al., 2020a; Weller et al., 2020b). Weather data for each sampling visit, and the preceding 30 days were downloaded from the NEWA station (newa.cornell.edu) closest to the sample site as previously described Weller et al. (2020a), Weller et al. (2020b), Weller et al. (2020c), while spatial data were downloaded from publicly available sources (see Supplementary Table S1). Average air temperature and solar radiation, and total rainfall were calculated for 0–1, 1–2, 2–3, 3–4, 4–5, 5–10, 10–20, and 20–30 days before sample collection. All spatial analyses were performed using ArcGIS version 10.2 or R version 3.5.3. The inverse-distance weighted percentage of each land cover class 1) in the whole watershed, 2) within the stream corridor (i.e., within 60 m of the stream channel), and 3) in the flood plain was calculated as previously described (King et al., 2005). In addition to characterizing land cover, we also determined if potential point sources of contamination were present upstream of each site as well as the density of these point sources (Supplementary Table S1). Summaries of these features in the training and test data (e.g., ranges, average values) can be found in the supplemental material of Weller et al. (2020a) and Weller et al. (2020b), respectively.

All samples were stored at 4°C and processed <18 h after collection. During processing, each 10-L GS was filtered through a modified Moore swab (mMS; (Sbodio et al., 2013)). After filtration, each mMS was transferred to a sterile Whirl-Pak and processed as described previously (see github.com/wellerd2/Laboratory-Protocols for the protocol). Briefly, 225 ml of buffered *Listeria* enrichment broth (BLEB; Becton Dickinson, Franklin Lakes, NJ) was added to each Whirl-pak. After incubating at 30°C for 4 h, *Listeria* selective enrichment supplement (Oxoid, Cambridge, United Kingdom) was added. After incubation for a total of 24 and 48 h at 30°C, 50 μl of enrichment were streaked onto *L. monocytogenes* plating medium (LMPM; Biosynth International, Itasca, IL) and Modified Oxford agar (MOX; Becton Dickinson). The LMPM and MOX plates were incubated for 48 h at 35 and 30°C, respectively. Up to 4 presumptive *Listeria* colonies were sub-streaked from MOX to LMPM. After these LMPM plates were incubated at 35°C for 48 h, up to 2 presumptive *L. monocytogenes* (blue on LMPM) colonies and up to 2 presumptive nonpathogenic *Listeria* spp (white on LMPM) colonies were selected for confirmation by amplification and sequencing of the partial *sigB* gene (Nightingale et al., 2005; Den Bakker et al., 2010; Bundrant et al., 2011). It is important to note, that for ~15% of samples in the training dataset, only 9 L were processed as described above; the remaining liter was filtered through a 0.45 um filter. The filter was then transferred to a sterile Whirl-pak bag, and processed using a modified version of the protocol above (i.e., using 90 instead of 225 ml of BLEB). For this subset of 2018 samples, if either the mMS or 0.45 um filter were confirmed as *Listeria* spp (excluding *L. monocytogenes*) or *L. monocytogenes*-positive than the sample was considered positive for the given target.

### Statistical Analyses

All analyses were performed in R (version 3.5.3; R Core Team, Vienna, Austria). Baseline models were created using existing water quality standards (Environmental Protection Agency, 2012; US FDA, 2015). Since each standard is based on an acceptable level of *E. coli* being present in the sample, samples with *E. coli* levels below this level were predicted to be negative for the target (*Listeria* spp. excluding *L. monocytogenes* or *L. monocytogenes*), while samples above this level were predicted to be positive. It is important to note that since *L. ivanovii* was not isolated in the present study, *Listeria* spp. excluding *L. monocytogenes* will henceforth be referred to as nonpathogenic *Listeria* spp. The cut-offs considered were: 126, 235, and 410 MPN of *E. coli/*100-ml (Environmental Protection Agency, 2012; US FDA, 2015). The epiR and exact2×2 packages were used to calculate performance measures for each baseline model. Boxplots were used to visually compare *E. coli* levels between *Listeria* positive and negative samples in the training and test data.

### Predictive Models

The 15 learners used in the present study were selected to ensure comparability with previous studies focused on predicting foodborne pathogen presence in preharvest environments i.e., [(Strawn et al., 2013; Golden et al., 2019; Polat et al., 2019; Weller et al., 2020c)]. All models were trained using the 2018 dataset Weller et al. (2020a), and tested using 2017 dataset (Weller et al., 2020b). Separate models were developed to predict the presence of nonpathogenic *Listeria* spp., and *L. monocytogenes*. Hyperparameter tuning was performed to maximize area under the curve (AUC) *via* repeated 3-fold cross-validation. After model tuning and training, predictive performance was assessed using the test data. By using the 2018 data to train the models and the 2017 data to test the models, the impact of overfitting on performance estimates was reduced. The probability threshold was tuned to maximize kappa score, since the values of several performance measures (e.g., sensitivity) are dependent on this threshold. Prior to model development, the training and test data were merged, and all features were centered and scaled. The training and test data were then split into separate datasets. Studies focused on developing deployable, field-ready models (i.e., models that can be used to build tools, such as smartphone-based applications, that growers can use to guide on-farm decision-making) should center and scale the training data, and then use the means and standard deviations from the training data to center and scale the test data.

**FIGURE 1 |:**
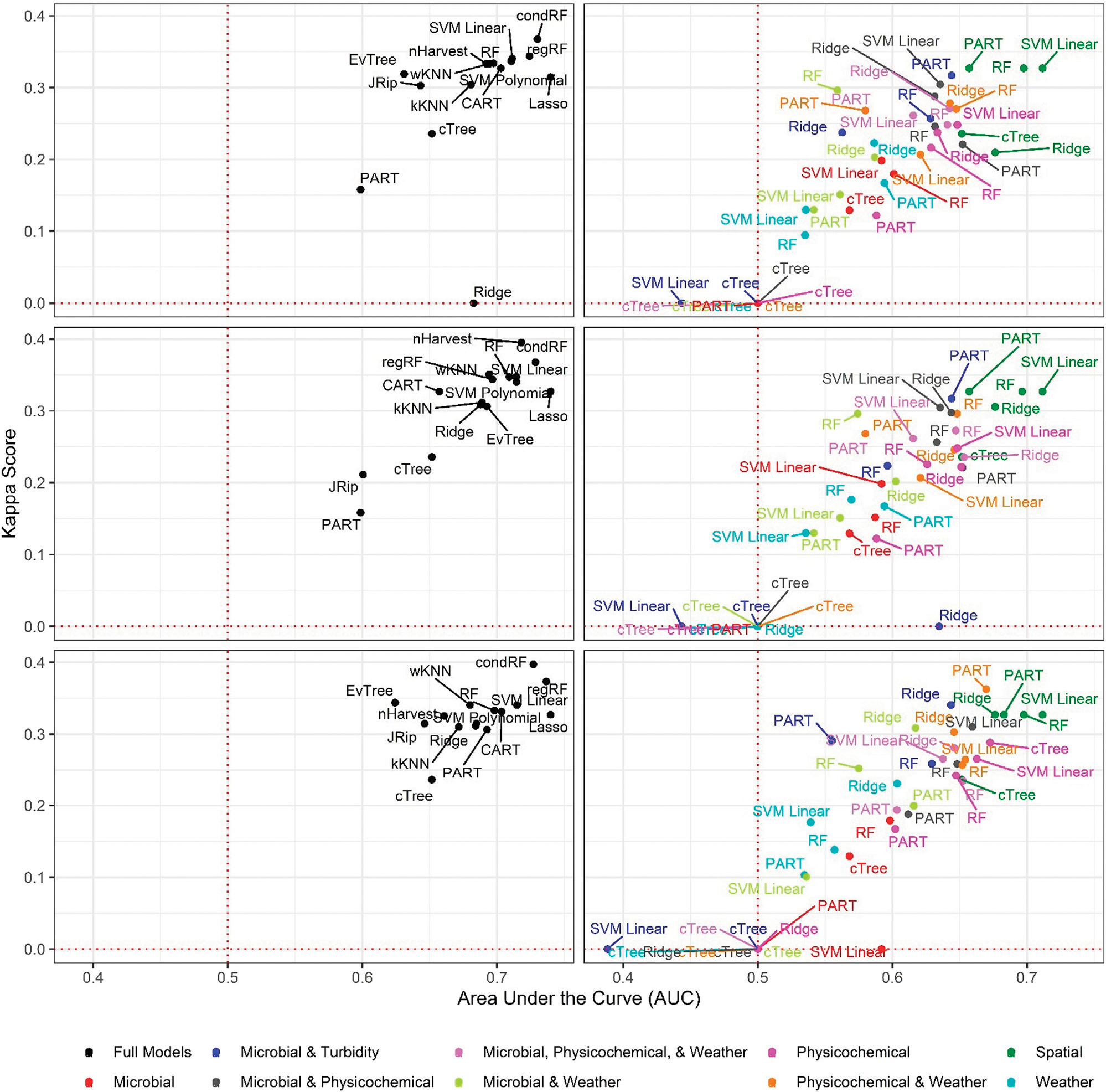
Area under the curve (AUC) and Kappa score for models that predict nonpathogenic *Listeria* spp (excluding *L. monocytogenes*) presence in New York agricultural water. To facilitate readability models are faceted into full (left column) and nested (right column) models, and by resampling method [no resampling (top row), oversampling (middle row), and SMOTE (bottom row)]. The dotted red lines indicate the cut-offs for AUC (0.50) and Kappa score (0.00) below which model’s performance is no better than chance.

**FIGURE 2 |:**
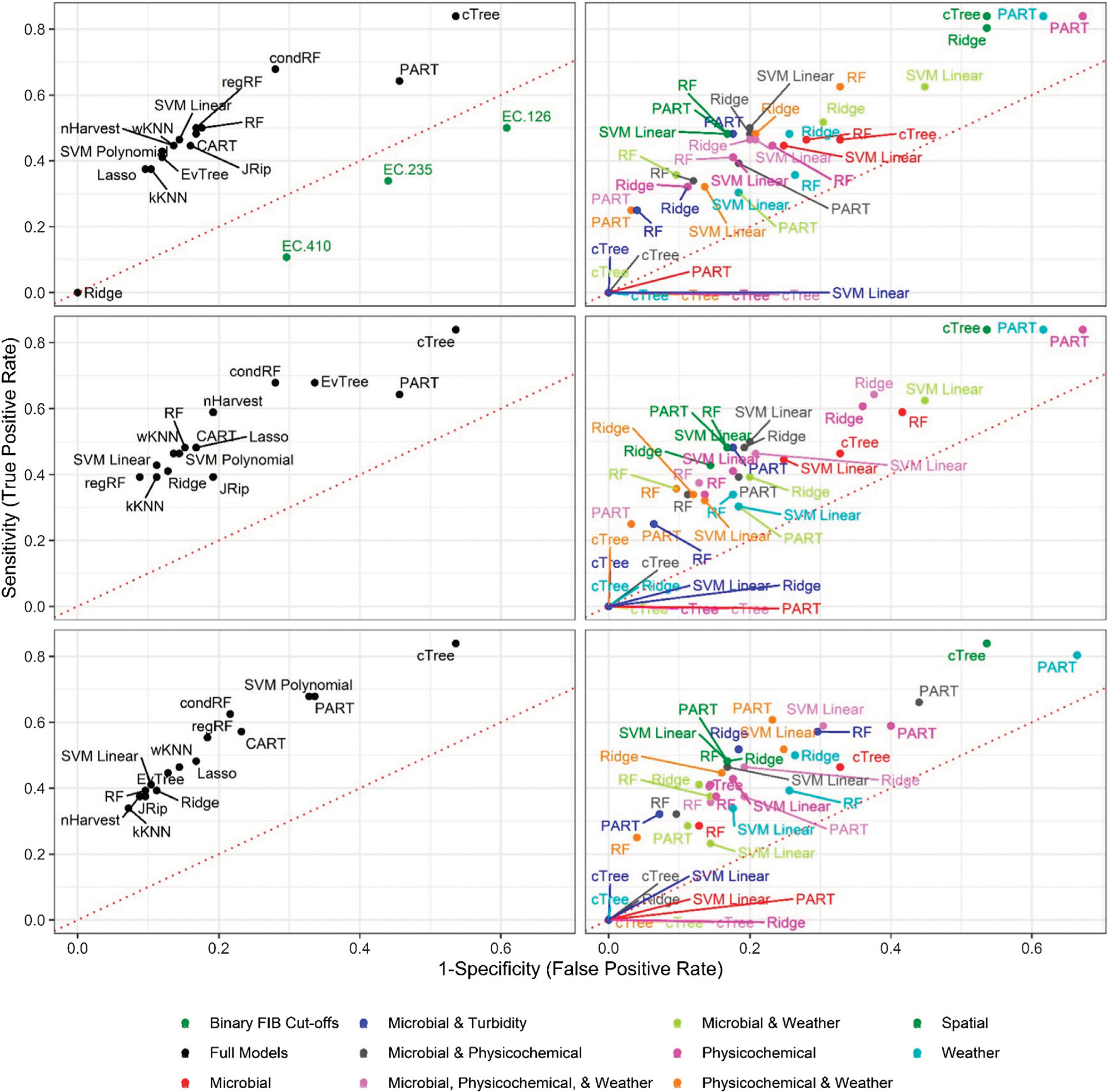
Sensitivity and 1-Specificity for models that predict nonpathogenic *Listeria* spp. (excluding *L. monocytogenes*) presence in New York agricultural water. To facilitate readability models are faceted into full (left column) and nested (right column) models, and by resampling method [no resampling (top row), oversampling (middle row), and SMOTE (bottom row)]. The dotted red lines represent perfect chance (i.e., a model that falls on the line has the same odds of correctly predicting nonpathogenic *Listeria* spp. status as an unbiased coin toss).

**FIGURE 3 |:**
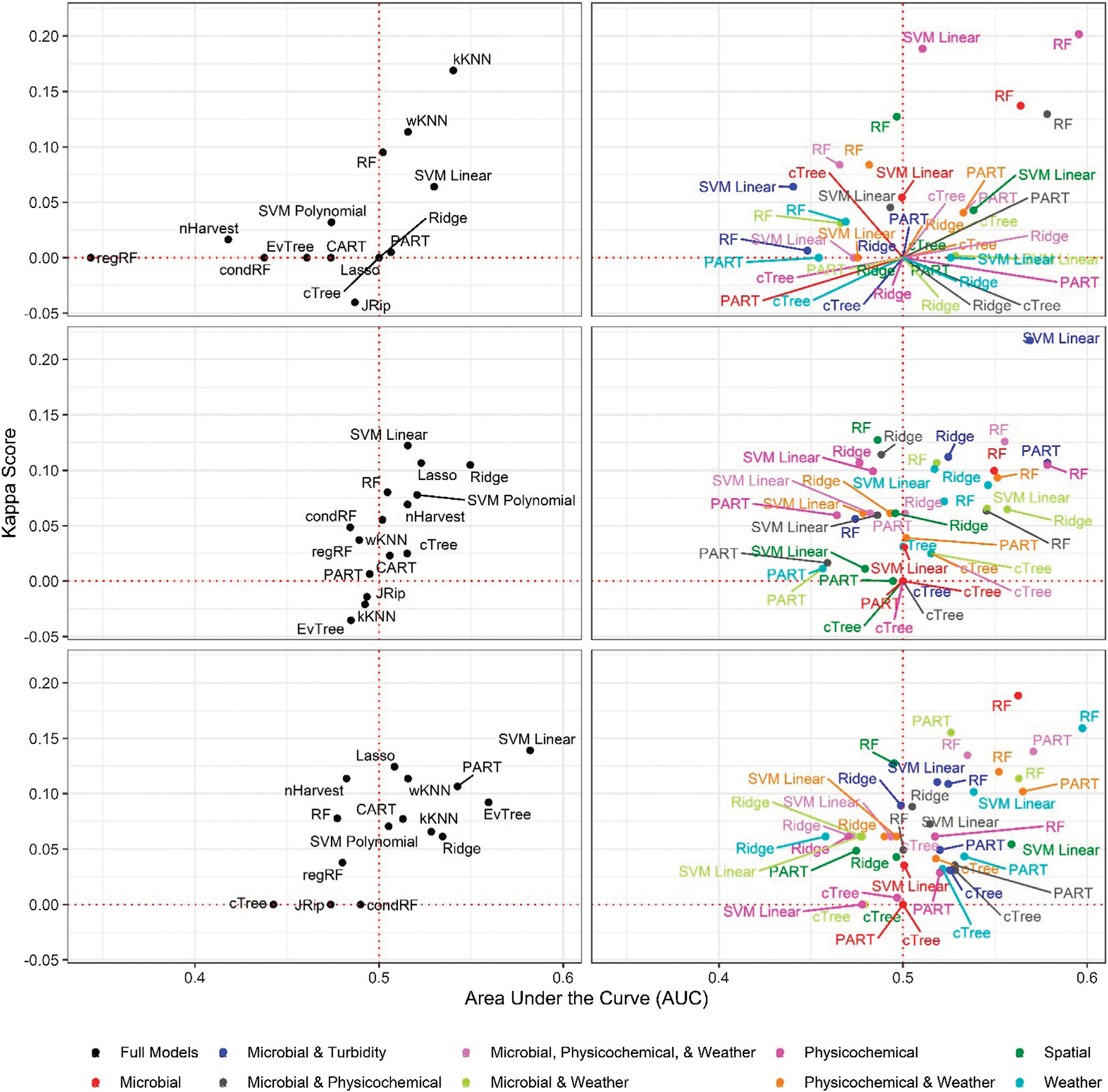
Area under the curve (AUC) and Kappa score for models that predict *L. monocytogenes* presence in New York agricultural water. To facilitate readability models are faceted into full (left column) and nested (right column) models, and by resampling method [no resampling (top row), oversampling (middle row), and SMOTE (bottom row)]. The dotted red lines indicate the cut-offs for AUC (0.50) and Kappa score (0.00) below which model’s performance is no better than chance.

**FIGURE 4 |:**
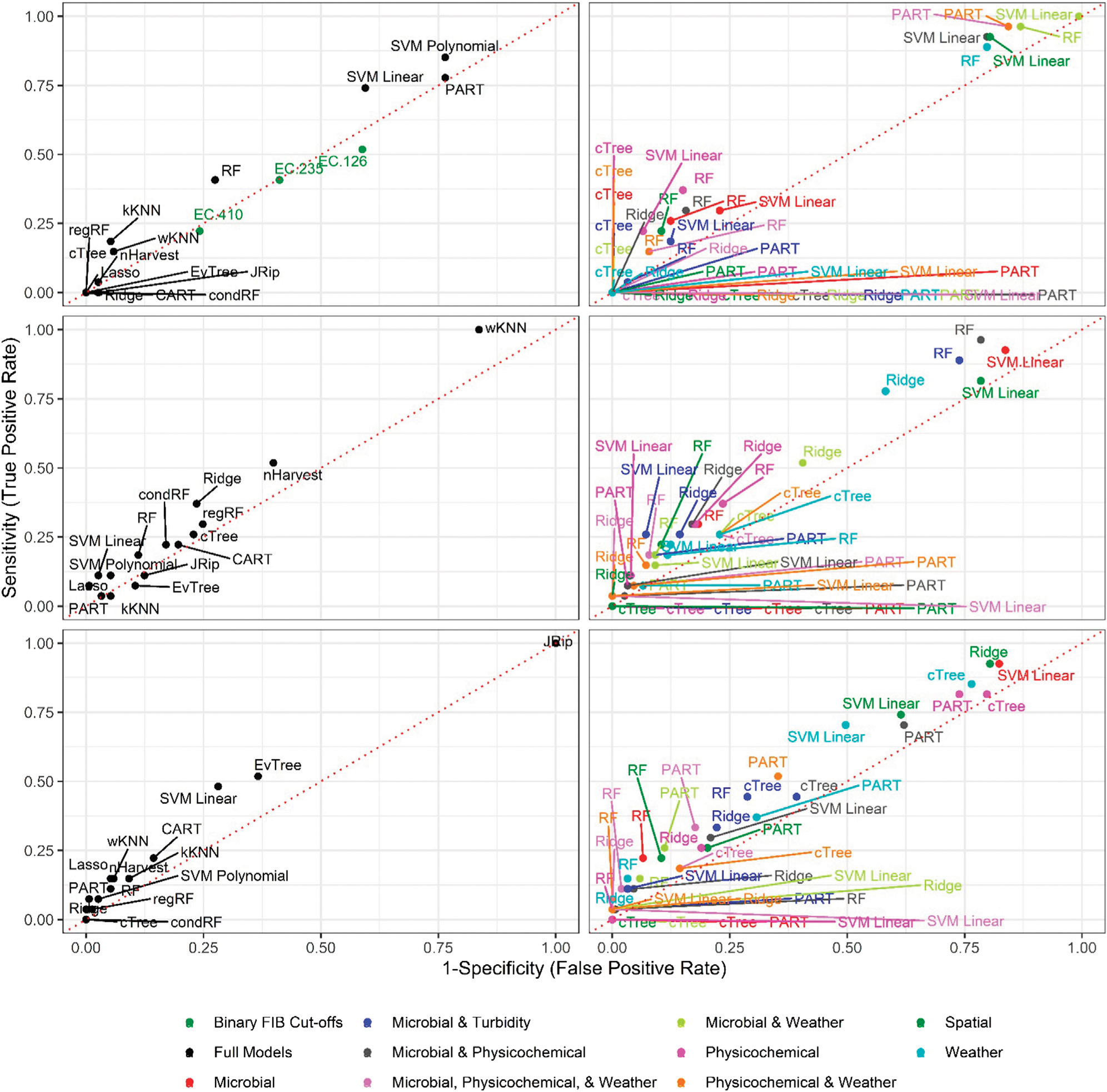
Sensitivity and 1-Specificity for models that predict *L. monocytogenes* presence in New York agricultural water. To facilitate readability models are faceted into full (left column) and nested (right column) models, and by resampling method [no resampling (top row), oversampling (middle row), and SMOTE (bottom row)]. The dotted red lines represents perfect chance (i.e., a model that falls on the line has the same odds of correctly predicting *L. monocytogenes* status as an unbiased coin toss).

During model development, between one and four types of features were used: 1) microbial water quality features; 2) physicochemical water quality features and temperature collected at the time of sample collection; 3) spatial features based on data extracted or calculated using geographic information systems; and 4) weather features based on weather data obtained from stations between <1 and 26 km from the sampling site [see Supplementary Table S1 for a complete list of each feature type; see (Weller et al. (2020a), Weller et al. (2020b) for summaries for the feature data]. Models built using all four feature types were designated “full models.” The 15 learners used to build the full models can be grouped into 1) tree-based learners, 2) ensemble learners (or forests), 3) regression, rule-based learners, and 4) support vector machines [SVM; for descriptions of each learner as well as its (dis)advantages and tunable parameters see (Bischl et al., 2016b; Kuhn and Johnson, 2016; Weller et al., 2020c; Weller et al., 2021)]. Separately from the full models, “nested models” were developed to assess the relative information gain associated with using different feature types for model training. Five of the 15 learners used to build the full models were selected to build the nested models. Nine nested models were then built for each of these five learners using between one and three of the feature types (see Supplementary Tables S2–S3; Figures 1–4). Performance measures for each model were calculated and visualized graphically. The top-ranked models for each outcome were identified by 1) ranking models based on AUC, F1-score, and kappa score, and 2) averaging each model’s rank for these 3 measures. A larger rank indicates better performance; models that tied were assigned the same rank. The performance of the top-ranked models for each outcome was visualized using density, ROC, and split quantiles plots. Explanations on how to interpret these plots are included in the figure legends.

Since the prevalence of nonpathogenic *Listeria* spp. and *L. monocytogenes* was below 30% in the training data, the training data was considered imbalanced (Table 1); specifically only 10 and 28% of the samples included in the training data were positive for *L. monocytogenes* and non-pathogenic *Listeria* spp., respectively (Table 1). As a result, the *L. monocytogenes* can be considered severely imbalanced and the non-pathogenic *Listeria* spp. data can be considered slightly imbalanced. Depending on the learner used, imbalanced outcomes data can result in inaccurate models that are biased toward the dominant class (e.g., the resultant model may call all novel samples *L. monocytogenes*-negative since that is the dominant class in the training data). Two ways for correcting this imbalance were considered here, oversampling and synthetic minority oversampling technique (SMOTE); models were also run without correcting for imbalance and are referred to as no resampling models (Chawla et al., 2002; Bischl et al., 2016b). Briefly, oversampling randomly duplicates (with replacement) samples representing the minority class (i.e., *Listeria*-negative samples), while SMOTE generates novel observations of the minority class. SMOTE works by randomly selecting an existing sample with the minority class, and then interpolating the feature data for this observation and its next nearest neighbors to create a new “novel” observation. While other approaches (e.g., undersampling, which eliminates majority class observations from the dataset) exist, they were not considered here due to the nature of the training dataset (e.g., the small sample size makes undersampling impractical). For each learner-feature set combination, separate models were built using each approach for addressing data imbalance. In oversampling, samples with the minority class value (i.e., nonpathogenic *Listeria* or *L. monocytogenes* positive samples) are randomly selected with repetition and added to the dataset until the prevalence of the minority class equals 30%.

## RESULTS AND DISCUSSION

The present study used existing datasets to train Weller et al. (2020a) and test Weller et al. (2020b) models to predict the probability of *Listeria monocytogenes* and nonpathogenic *Listeria* spp (i.e., *Listeria* spp. excluding *L. monocytogenes*) being present in streams used to source water for produce production in New York state. Given the imbalanced nature of the training data (Table 1), one aim of the current study was to assess the impact on predictive performance of different methods for dealing with imbalanced training data. The other study aim was to generate information that can guide future efforts on how machine learning approaches can be used to develop models to predict foodborne pathogen presence in agricultural water (e.g., feature types and learners that future models should focus on). It is therefore important to note that the models developed here are not field-ready models (i.e., models that can be used on-farms to guide risk mitigation efforts), and instead provide a framework that future studies can build upon to develop field-ready models. It is important to note that 1) these future efforts should focus on pathogens of greatest concern in agricultural water (i.e., EHEC and *Salmonella*) and 2) nonpathogenic *Listeria* spp. and *L. monocytogenes* were used here because we lacked access to suitable *Salmonella* and pathogenic *E. coli* data. However, *L. monocytogenes* is a foodborne pathogen of concern and *L. monocytogenes* contamination of agricultural water may lead to human illness.

### Baseline Models Built using Binary *E. coli* Cut-offs were Unable to Predict *Listeria*-Positive or *Listeria*-Negative Samples

In total, we developed 6 baseline models [2 microbial targets*3 *E. coli* cut-offs], 90 full models [2 microbial targets*15 learners*3 resampling approaches] and 268 nested models [2 microbial targets*5 learners*3 resampling approaches*9 feature sets]. The three resampling approaches used were 1) no resampling (i.e., the raw data were used), 2) SMOTE resampling, and 3) oversampling. The three baseline models were unable to accurately differentiate *L. monocytogenes* or non-pathogenic *Listeria* spp. positive samples from *Listeria* negative samples. (Figures 1–4). The best performing *L. monocytogenes* and non-pathogenic *Listeria* spp. baseline models, which used the 410 CFU/100-ml cut-off, were ranked 81^st^ and 65^th^, respectively, while the top-performing full/nested models were ranked 176^th^ and 181^st^, respectively (Table 2, Supplementary Tables S2–S3; note, a larger rank indicates better performance). For all baseline models, performance measures were equal to or below the cut-offs (e.g., a AUC of 0.50) used to identify models that outperformed a random classifier (Table 2). Although performance measures were calculated using the test dataset, the baseline models performed similarly when predictions were made on the training data (Supplementary Figure S1), indicating that using binary *E. coli* cut-offs to assess if *Listeria* was potentially present in agricultural surface water is not effective. This result is not surprising since multiple studies have found that agricultural and recreational water standards based on binary *E. coli* cut-offs were insufficient for assessing food safety hazards in surface waterways (Thoe et al., 2014; Havelaar et al., 2017; Truitt et al., 2018; Weller et al., 2020c). The poor performance of the baseline *E. coli* models in the present study is also consistent with the conceptual basis behind *E. coli-*based monitoring programs. Specifically, *E. coli* is an indicator of fecal contamination, and its use is predicated on the assumption that foodborne pathogen in agricultural water are of fecal origin (Busta et al., 2006; Chapin et al., 2014; Uyttendaele et al., 2015). However, *L. monocytogenes* is an opportunistic pathogen, and both *L. monocytogenes* and nonpathogenic *Listeria* species exist as free-living populations in non-host environments [e.g., soil, water; (Vivant et al., 2013)]. As such, fecal indicators, like *E. coli*, may be ill-suited to assessing the potential presence of *Listeria* in surface water. Overall, the findings of this and other studies [e.g., Havelaar et al. (2017), Truitt et al. (2018)] are illustrative of the need for alternative or supplementary strategies to existing *E. coli*-based monitoring programs for assessing and managing food safety hazards in surface water used for produce production (e.g., predictive model-based applications), particularly for microbial hazards that are not predominantly fecal in origin. Moreover, the fact that the machine learning models outperformed the baseline *E. coli* models in the present study suggests that machine-learning models may be useful for predicting when and where *Listeria* is likely to be present in surface water used for produce production.

### While Ensemble and Black-box Learner Performance was Robust to Resampling for Moderately Imbalanced Data, Models Built using Synthetic Minority Oversampling (SMOTE) Outperformed, on Average, Models Built using Oversampling or without Resampling

Six of the top-ranked non-pathogenic *Listeria* spp. models were built using SMOTE resampling, two were built without resampling (i.e., where imbalance was not corrected), and one was built using oversampling. On average, nonpathogenic *Listeria* spp. models built without resampling performed worse than models built using the same learner and features (i.e., paired models), but using SMOTE or oversampling (Figures 1, 2; Supplementary Table S3). When paired, nonpathogenic *Listeria* spp. models were compared, the SMOTE models outperformed the oversampling or no resampling models 43% of the time, the oversampling models outperformed the SMOTE and no resampling models 25% of the time, and the no resampling models only outperformed the resampling models 6% of the time (Supplementary Table S3). It is important to note that, the performance measures for 14 nonpathogenic *Listeria* spp. models, including two of the top-ranked models, were the same regardless of resampling approach used (Table 2). These ties indicate that, regardless of the resampling method, the same model was generated; as such, these learners appear invariant to data imbalance. Indeed, the effect of resampling, as evidenced by differences in paired model rankings, was more pronounced for nonpathogenic *Listeria* spp. models built using tree-based learners compared to models built using ensembles (e.g., forests), black-box learners (e.g., SVMs), or regression learners (Figures 1, 2; Table 2; Supplementary Table S3). Similarly, the effect of resampling was more pronounced for nested models built using individual feature types compared to full models or nested models built using multiple feature types (Figures 1, 2; Table 2; Supplementary Table S3). For the *L. monocytogenes* models, four of the top-ranked models were built without resampling, four were built using SMOTE resampling, and two were built using oversampling (Table 2). However, models built without resampling performed substantially worse compared to SMOTE and oversampling models overall (Figure 3; Supplementary Table S2). In fact, when paired models were compared, the no resampling models ranked higher than the resampling models only 10% of the time, while the SMOTE models were ranked highest 57% of the time. Similarly, models with no resampling accounted for 53% of *L. monocytogenes* models that performed the same as or worse than random classifiers, while the oversampling and SMOTE models only accounted for 31 and 16%, respectively, of these models. Thus, just like the nonpathogenic *Listeria* spp. models, using SMOTE to address data imbalance appeared to produce better performing *L. monocytogenes* models (Figures 3, 4; Table 2; Supplementary Table S2).

It is important to note that the impact of not resampling was substantially higher for the *L. monocytogenes* models compared to the nonpathogenic *Listeria* spp. models (Figures 1, 3). Twenty-seven *L. monocytogenes* models built without resampling performed worse than a random classifier compared to only seven nonpathogenic *Listeria* spp. models built without resampling (Supplementary Tables S2–S3). Since the *L. monocytogenes* data were severely imbalanced and the nonpathogenic *Listeria* spp. data were only slightly imbalanced, such a finding is logical, since it is well-established that the degree of training data class imbalance affects model performance (Japkowicz, 2000; Bischl et al., 2016a; Kuhn and Johnson, 2016). In fact, resampling approaches were developed to overcome this phenomenon. Although our finding that SMOTE models, on average, outperformed oversampling models may be specific to our dataset, oversampling replicates existing cases in the training data and can thus cause overfitting (Batista et al., 2004; Chawla et al., 2004). Overfitting produces a model that can describe the training data well but is not generalizable to novel datasets; an independent test dataset was used here to minimize the impact of this when calculating performance measures. Since SMOTE resampling was developed to overcome this and other limitations of oversampling Chawla et al. (2002), Fernández et al. (2018), it is unsurprising that SMOTE outperformed oversampling in the present study. Overall, our findings suggest that future projects aimed at developing deployable models to predict pathogen presence in agricultural water should implement resampling to address class imbalance. When considering how to implement resampling in these future studies, it is should be noted note that the effect of resampling on model performance may be learner specific (Prati et al., 2015). For example, Prati et al. (2015), found that the impact of class imbalance on model performance was dependent on the learner used, with SVMs being the least affected by class imbalance and rule-based learners, like JRip, being the most affected. This finding is consistent with the results of the present study, where the effect of class imbalance was more pronounced for nonpathogenic *Listeria* spp. models built using tree-based learners compared to ensemble, black-box and regression learners. Thus, despite the fact that in our study SMOTE resampling appears to produce better performing models compared to oversampling, additional research is needed to confirm this conclusion. As such, future studies may want to consider learners whose performance appears relatively invariant to resampling method (e.g., SVMs as opposed to JRip).

### While Random Forests Outperformed all Other Learners, SVMs Models were also Consistently Ranked Among the Top-Performing Models Regardless of Resampling Approach used

Random forests accounted for 6 and 3, respectively, of the ten top-ranked nonpathogenic *Listeria* spp. and *L. monocytogenes* models (Table 2). One SVM model, two PART models, and 2 regression models were also among the 10 top-ranked nonpathogenic *Listeria* spp. models, while 3 SVM models, 1 PART, and 1 regression model were also among the top-ten *L. monocytogenes* models (see Table 2 for all 10 top-ranked models). Visual inspection of the graphs comparing nonpathogenic *Listeria* spp. model performance shows random forest, SVMs, and regression models consistently clustering in the top right of Figure 1, and in the top left of Figure 2, indicating good performance regardless of resampling method or feature types used in model development. While SVM, regression, and nested random forest models for *L. monocytogenes* cluster in the top right of Figure 3 (indicating better performance compared to the other *L. monocytogenes* models), overall poor model performance (as indicated by the number of models with AUC < 0.50) prevents drawing a definite conclusion based on comparisons between learners for the *L. monocytogenes* models. Despite this limitation, we can conclude that for both the nonpathogenic *Listeria* spp. and *L. monocytogenes* models, models built using the cTree, JRip, and CART learners were consistently among the worst performing models (Figures 1, 3). Overall, these findings are consistent with past studies that compared the ability of models built using different learners to accurately predict pathogen presence in farm and freshwater environments (Pang et al., 2017; Avila et al., 2018; Golden et al., 2019; Weller et al., 2020c). For example, one study Weller et al. (2020c) that compared the ability of models built using different learners to predict enteric pathogen presence in agricultural water also found that forest and SVM learners outperformed other learner types. Similarly, a study, that developed models to predict *Listeria* presence in feces and soil collected from pasture poultry farms, compared the performance of boosted forests and random forests and showed that good predictive accuracy (AUC between 0.7 and 0.9) could be achieved using forest-based learners (Golden et al., 2019). Another study that used regression and random forest to characterize associations between weather conditions and *Listeria* presence in environmental samples collected from a mixed produce-dairy farm found that both regression and random forest models performed well with AUCs between 0.80 and 0.92 (mean = 0.83), and 0.70 and 0.88 (mean = 0.80), respectively (Pang et al., 2017). In general, random forest and SVM algorithms are more robust to missingness and correlation/collinearity between features, and are better able to account for complex relationships between multiple features (e.g., interaction, hierarchical relationships) than regression methods. Overall, our findings suggest that future studies focused on developing deployable models to predict pathogen contamination in surface waterways used to source water for produce production should focus model development efforts on random forest, SVM, or regression learners but not tree- or rule-based learners. Moreover, since ecological datasets often suffer from data complications (e.g., missing data due to breaking probes, storms, etc.) and past studies have shown that complex interactions between environmental and anthropogenic features impact microbial water quality (Wilkes et al., 2009; Bradshaw et al., 2016; Weller et al., 2020b), SVMs and random forest learners may be better suited to the development of deployable models than regression learners.

### Models Built using Spatial Features Outperformed Models Built using Other Feature Types When Predicting Nonpathogenic *Listeria* Spp.

**FIGURE 5 |:**
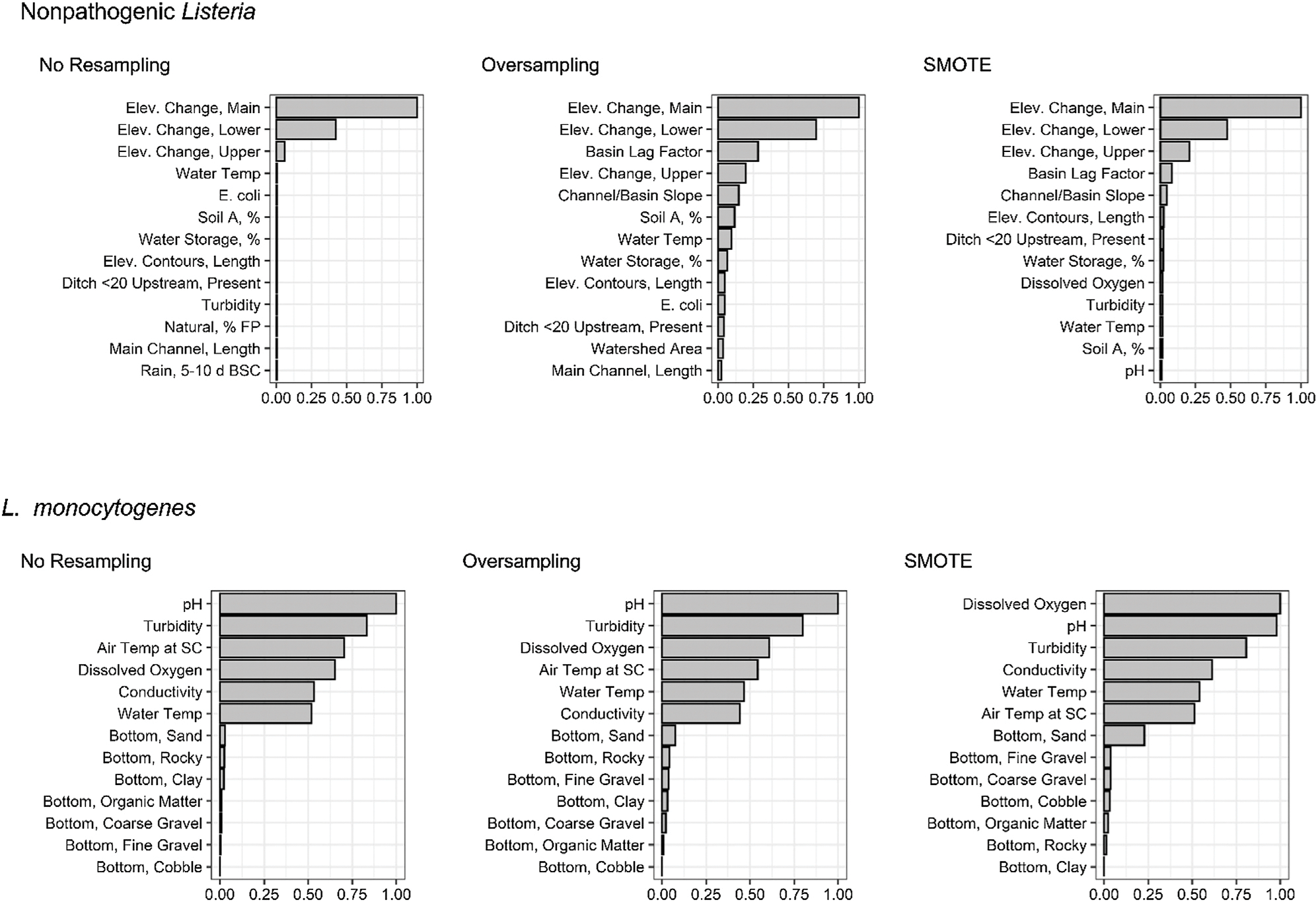
Variable importance plots showing the ranking of each feature used to develop the 1) full nonpathogenic *Listeria* spp (excluding *L. monocytogenes*) conditional forest models since the SMOTE model was the top-ranked nonpathogenic *Listeria* spp. model, and 2) nested, physicochemical *L. monocytogenes* random forest models since the no resampling model was the top-ranked *L. monocytogenes* model; Table 2). The *x*-axis of each plot is normalized variable importance (VI), and the features on the *y*-axis are arranged from greatest impact (higher VI) to lowest impact (lower VI) on predictive accuracy. For the nonpathogenic *Listeria* spp. forests, only the thirteen top-ranked features are shown to facilitate readability. Bottom, stream bottom substrate; Elev., elevation; Soil A, hydrologic soil type-A.

When resampling method is ignored, certain patterns become evident amongst nested models (Supplementary Figure S6). For instance, the spatial nonpathogenic *Listeria* spp. models consistently outperformed the nested models built using microbial, weather, and physicochemical water quality (Figures 1, 2). In fact, regardless of resampling method, when features were ranked by impact on predictive accuracy (using permutation variable importance) in the top-ranked, non-pathogenic *Listeria* spp. full model, 9 of the 13 features with the greatest impact were spatial features associated with topography, soil type and run-off potential, watershed size, and potential sources of contamination (Figure 5). Such findings are consistent with past studies that found strong associations between spatial factors and pathogen contamination of both surface water and produce preharvest environments (Ivanek et al., 2009; Strawn et al., 2013; Chapin et al., 2014; Linke et al., 2014; Stea et al., 2015; Falardeau et al., 2017; Harrand et al., 2020). For instance, Stea et al. (2015) compared *Listeria* spp. contamination in two Nova Scotia, Canada watersheds, and found that *Listeria* spp. was more prevalent in rural (38%) than urban (22%) watersheds. Similar to the study reported here, Ivanek et al. found that topographical factors were associated with *Listeria* spp. isolation from water samples in New York natural environments (Ivanek et al., 2009).

Unlike the nonpathogenic *Listeria* models, the spatial *L. monocytogenes* nested models consistently performed equal to or worse than random, suggesting that, for the streams represented in the test data, use of spatial features was not informative when predicting *L. monocytogenes* presence. Instead, weather and physicochemical water quality parameters appeared to drive predictive accuracy for the *L. monocytogenes* models (Figure 5). However, this may be an artifact of the data used here. Specifically, 1) the training data were collected from a large number of sites (N = 68) visited 2–3 times each, 2) the test data were collected from six sites each visited 15 to 34 times, and 3) the fact that the microbial water quality has been shown to vary considerably over small spatial scales for the sampled streams (Weller et al., 2020a; Weller et al., 2020b). These three factors, when coupled with the low prevalence of *L. monocytogenes* detection (Table 1), means that spatial signals could be missed since combinations of spatial and temporal features that facilitate *L. monocytogenes* contamination of surface water (e.g., being downstream of a pasture during a rain event) may not be represented in the test dataset. It is important to note that these limitations are less likely to affect the nonpathogenic *Listeria* results due to the greater prevalence of nonpathogenic *Listeria* in the present study (Table 1). To overcome these limitations, future studies that aim to develop and validate deployable models for *L. monocytogenes* should ensure that, for both the training and test data, 1) sufficient sites are sampled to capture variation in spatial factors even when prevalence is low (e.g., 10%), 2) each site is sampled with sufficient frequency to capture rare, stochastic contamination events, 3) the sampling sites are representative of agricultural waterways in the given region, and 4) sufficient samples are collected so, even if pathogen prevalence is low, there are sufficient positives to capture relevant spatial and temporal signals.

## CONCLUSION

The findings from this study are consistent with past studies focused on predicting enteric pathogen presence in agricultural water Polat et al. (2019), Weller et al. (2020c), fecal indicator levels in agricultural water Buyrukoğlu et al. (2021), Weller et al. (2021), and *Listeria* presence in preharvest environments (Strawn et al., 2013; Weller et al., 2016; Pang et al., 2017; Golden et al., 2019). Our findings and those of these previous studies indicate that machine learners can be used to develop models that accurately predict pathogen presence in agricultural water. In fact, our findings suggest that predictive models were able to more accurately assess *Listeria* contamination status for New York stream than models created using existing *E.* coli-based water quality standards. As such, predictive models could be incorporated into on-farm risk management plans and used to help growers make evidence-based risk management decisions in real-time. However, the present study isa proof-of-concept study aimed at addressing three key knowledge gaps surrounding 1) the utility of predictive models for assessing *Listeria* contamination risks in streams that provide water for produce production, 2) the utility of different strategies for addressing class imbalance when developing these *Listeria* models, and 3) the type of features that should be used when developing these *Listeria* models. As such, future studies are needed to develop robust, deployable models that can be used to guide on-farm decision-making. Our findings provide guidance on the how future studies can develop these models; these studies will require substantially larger datasets than the dataset used in the study presented here. Specifically, our findings suggest that using SMOTE resampling to address class imbalance will result in more accurate models as will the use of random forest learners. While our findings suggest that spatial features are uninformative when assessing *L. monocytogenes* risks, this may be an artifact of the sampling strategy used when collecting the training and test data. Given the fact that spatial features were more informative than any other feature type for predicting nonpathogenic *Listeria* spp. presence, future studies should still consider spatial features when developing models to predict *L. monocytogenes* in agricultural water. Conversely, physicochemical water quality parameters were strongly associated with accurately predicting *L. monocytogenes* presence in the current study and should be included as features in future studies focused on developing deployable *L. monocytogenes* models.

## Supplementary Material

Data Sheet 1

The Supplementary Material for this article can be found online at: https://www.frontiersin.org/articles/10.3389/fenvs.2021.701288/full#supplementary-material

## Figures and Tables

**TABLE 1 |:** *L. monocytogenes* and *Listeria* spp. (excluding *L. monocytogenes*)^a^ prevalence in the training and test datasets.

Dataset	No. of		Prevalence (no. Pos. Samples/Total samples)		Year (citation)
	Sampling sites	Visits per site	*L. monocytogenes*	Nonpathogenic *Listeria* spp.	

Training	68	2–3	10% (20/191)	28% (53/191)^b^	2018 (Weller et al., 2020a)
Test	6	15–34	15% (27/180)	31% (55/180)^c^	2017 (Weller et al., 2020b)

a *Since* L. ivanovii *was not isolated here*, Listeria *spp. (excluding* L. monocytogenes*) is referred to as nonpathogenic Listeria spp. throughout the paper*.

b *The following Listeria species were detected and included in this composite category:* L. innocua *(11/191),* L. marthii *(5/191),* L. seeligeri *(21/191), and* L. welshimeri *(16/191)*.

c *The following Listeria species were detected and included in this composite category:* L. innocua *(9/180),* L. marthii *(11/180),* L. rustica *(1/180),* L. seeligeri *(25/180), and* L. welshimeri *(15/ 180). Note that several samples tested positive for multiple nonpathogenic Listeria species, which did not occur in the test data.*

**TABLE 2 |:** Summary of the top-ranked full and nested models for predicting nonpathogenic *Listeria* spp (excluding *L. monocytogenes*) and *L. monocytogenes* presence in New York streams, including how these top-ranked models compared to baseline models created using existing water quality standards (EPA, 2012; US FDA, 2015). It is important to note that a higher model ranks indicates a better performing model. For example, the best-performing nonpathogenic *Listeria* spp. and *L. monocytogenes* models were ranked 181 and 176, respectively; the difference in the value of the top-ranked models for nonpathogenic *Listeria* spp. and *L. monocytogenes* is due to the fact that the models with a tied rank were assigned the same value.

Learner (features)	Resample approach^a^	Rank	AUC^b^	DOR^c^	Kappa^d^	MCC^e^

**Nonpathogenic *Listeria***						
Binary FIB cut-off models^f^						
126 MPB/100-ml	–	67^th^	–	0.7	−0.10	−0.09
235 MPB/100-ml	–	66^th^	–	0.6	−0.11	−0.10
410 MPB/100-ml	–	65^th^	–	0.3	−0.20	−0.20
10 Top-performing full and nested models^f^						
Conditional forest (full model)	SMOTE	181^th^	0.73	6.1	0.40	0.39
Conditional forest (full model)	None & over	180^th^	0.73	5.4	0.37	0.37
Node harvest (full model)	Over	179^th^	0.72	6.0	0.40	0.39
Regularized random Forest(Full model)	SMOTE	178^th^	0.74	5.5	0.37	0.37
Regularized random forest (full model)	None	170^th^	0.72	5.0	0.34	0.34
Partial decision Trees (nested model, physicochemical & weather)	SMOTE	162^nd^	0.67	5.1	0.36	0.36
SVM with linear hyperplane (nested model, spatial)	Tie^g^	152^nd^	0.71	4.6	0.33	0.32
Random forest (nested model, spatial)	Tie^g^	148^th^	0.70	4.6	0.33	0.32
Partial decision Trees (nested model, spatial)	SMOTE	143^rd^	0.68	4.6	0.33	0.32
Ridge regression (nested model, microbial & turbidity)	SMOTE	141^th^	0.64	4.8	0.34	0.34
Ridge regression (nested model, spatial)	SMOTE	141^th^	0.68	4.6	0.33	0.32
***Listeria monocytogenes***						
Binary FIB cut-off models						
126 MPB/100-ml	–	89^th^	–	0.8	−0.07	−0.05
235 MPB/100-ml	–	93^rd^	–	1.0	0.00	0.00
410 MPB/100-ml	–	81^st^	–	0.9	−0.02	−0.01
10 Top-performing full and nested models^f^						
Random forest (nested model, physicochemical)	None	176^th^	0.60	3.3	0.20	0.20
SVM linear (nested model, microbial & turbidity)	Over	173^rd^	0.57	4.5	0.22	0.23
SVM linear (full model)	SMOTE	172^nd^	0.58	2.4	0.14	0.15
Partial decision Trees (nested model, microbial, physicochemical, and weather)	SMOTE	165^th^	0.57	2.3	0.14	0.14
Random forest (nested model, microbial)	SMOTE	164^th^	0.56	4.1	0.19	0.19
Random forest (nested model, microbial & physicochemical)	None	159^th^	0.58	2.3	0.13	0.13
Evolutionary optimal Trees (full model)	SMOTE	152^nd^	0.56	1.9	0.09	0.11
K-nearest neighbor (full model)	None	149^th^	0.54	4.1	0.17	0.19
Ridge regression (full model)	Over	149^th^	0.55	1.9	0.10	0.11
SVM linear (full model)	None	139^th^	0.53	2.0	0.06	0.11

a To assess the effect of resampling on model performance, two resampling methods [oversampling (Over) and synthetic minority oversampling (SMOTE)] as well as no resampling (None) were used to develop three, separate models for each outcome-learner-feature type combination.

b Area under the Curve. AUC ranges between 0 and 1, with AUC = 1.0 indicating perfect prediction. AUC ≤ 0.5 indicates that the model’s performance is equal to or worse than chance.

c Diagnostic Odds Ratio. DOR ≤ 1 indicates a poor performing models (i.e., a model where a positive prediction is associated with a sample testing negative for the target microbe), while a DOR > 1 indicates that a positive prediction is associated with the sample testing positive for the target.

d Kappa score represents how much better the model performs compared to a model that randomly calls novel samples pathogen positive or negative. A score ≤ 0.0 indicates that the model is no better than random, while a score = 1 indicates a model that always correctly identifies novel samples.

e Matthew’s Correlation Coefficient. MCC ranges between–1 (the model always incorrectly predicts pathogen status) and 1 (the model always correctly predicts pathogen status). MCC ≤ 0.0 indicates that the model is no better than a model that randomly predicts pathogen status.

f To assess the relative information gain associated with using different feature types to build the models, two sets of analyses were performed. In the first set, each learner and the full set of features (Supplementary Table S1) were used to develop full models. In the second set, the features listed in Supplementary Table S1 were divided into four groups: microbial; physicochemical water quality and temperature data collected on site; weather data obtained from NEWA weather stations; and spatial. Nested models were then built using different combinations of these feature types.

g The performance measures for the models built using each of the three resampling methods for the given combination of learner and feature type were exactly the same. This indicates that regardless of the resampling method used the same model was generated.

## Data Availability

The data analyzed in this study is subject to the following licenses/restrictions: Data include confidential information and are available from the corresponding author on request. Requests to access these datasets should be directed to Daniel Weller at wellerd2@gmail.com or dlw263@cornell.edu.

## HIGHLIGHTS

- Pathogen contamination of water used for agriculture poses a public health risk
- Existing ag water quality standards (AWS) are insufficient for ensuring food safety
- Predictive models may provide an alternative to AWS for assessing hazards in water
- Data imbalance needs to be addressed when developing models for use in the field
- SMOTE resampling to address imbalance generated the most accurate predictive models
