# Supplementary material for: Comparison of Resampling Algorithms to Address Class Imbalance when Developing Machine Learning Models to Predict Foodborne Pathogen Presence in Agricultural Water
Source: Front Environ Sci. Author manuscript; Available in PMC 2026 Mar 19. (PMC12998587; doi:10.3389/fenvs.2021.701288)
Supplement: Data Sheet 1 [file NIHMS2130480-supplement-Data_Sheet_1.docx]

Table S1: Factors included in the analyses reported here. Values for all weather factors with the exception of temperature were calculated for 0-1, 1-2, 2-3, 3-4, 4-5, 5-10, 10-20 and 20-30 d before sample collection (BSC). Values for temperature were calculated for 0-5, 5-10, 10-20 and 20-30 d BSC due to the strong correlation between temperature 0-1, 1-2, 2-3, 3-4 and 4-5 d BSC.

| Factor | | | | Description | Data Type ^a^ | Date | Citations and Websites |
| --- | --- | --- | --- | --- | --- | --- | --- |
| Data Extracted from Publicly-Available Databases | | | | |  |  |  |
|  | Culverts: Carrying a waterway under a state highway and have a span of 5 to 20 feet | | | |  | 2014 | (*3*, *4*, *11*) |
|  |  | Present | | Were culverts present upstream? | S |  |  |
|  |  | Density | | Upstream density (no. of per 10 km^2^) | S |  |  |
|  | Dams | | |  |  | 2018 | (*5*) |
|  |  | Density | | Upstream density (no. of per 10 km^2^) | S |  |  |
|  | Road Crossing | | |  |  | 2015 | (*17*) |
|  |  | | Density | Upstream density (no. of per 10 km^2^) | S |  |  |
|  |  | | Min. Dist. | The flow path distance to nearest point upstream where a road crossed the stream (km). | S |  |  |
|  | Outfalls: Municipal stormwater outfalls along or near highways (presence is an indicator of urbanization) | | | |  | 2008 | (*6*, *7*, *12*) |
|  |  | Present | | Were stormwater outfalls present upstream? | S |  |  |
|  |  | Density | | Upstream density (no. of per 10 km^2^) | S |  |  |
|  | Municipal Wastewater Discharge Sites: Based on permits issued under the NYS^a^ Pollutant Discharge Elimination System (SPDES) | | | |  |  |  |
|  |  | Present | | Were wastewater discharge sites present upstream? | S |  |  |
|  |  | Density | | Upstream density (no. of per 10 km^2^) | S |  |  |
|  | Industrial Wastewater Discharge Sites: Based on permits issued under the NYS^b^ Pollutant Discharge Elimination System (SPDES) | | | |  | 2018 | (*15*) |
|  |  | Present | | Were wastewater discharge sites present upstream? | S |  |  |
|  |  | Density | | Upstream density (no. of per 10 km^2^) | S |  |  |
|  | In-stream Waterbodies: Bodies of water within the stream channel (e.g., mill ponds, impoundments, lakes) | | | |  | 2017 | (*8*) |
|  |  | Present | | Were waterbodies upstream? | S |  |  |
|  |  | Density | | Upstream density (no. of per 10 km^2^) | S |  |  |
|  | Septic System Density | | | Upstream density (no. per 10 km^2^). | S | 2011 | (*13*) |
|  | Solid Waste Site: Based on permits issued by NYS that allow application and spreading of manure, human septage, food processing, or other waste | | | |  | 2019 | (*14*) |
|  |  | Density | | Upstream density (no. of per 10 km^2^) | S |  |  |
|  | Land Cover ^c^ | | | |  | 2016 | (*9*, *10*) |
|  |  | Open Water | | Class 11 in National Land Cover Database (NLCD) | S |  |  |
|  |  | Cropland | | Cropland; Class 82 in NLCD | S |  |  |
|  |  | Pasture | | Pasture; Class 81 in NLCD | S |  |  |
|  |  | Developed | | Developed; Classes 21-24 in NLCD | S |  |  |
|  |  | For-Wet | | Natural cover; Classes 41-43, 51-52, 90, and 95 in NLCD (i.e., forest, shrubland or wetland) | S |  |  |
|  |  | Impervious | | Percent of upstream watershed that was under impervious cover | S |  |  |
|  | Watershed Area | | | Total area of upstream watershed (10-km^2^) | S | - | - |
|  | Stream Stats Data | | |  |  | 2019 | [streamstats.usgs.gov/ss/](https://streamstats.usgs.gov/ss/) |
|  |  | BSLOPCM | | Mean basin lope determined by summing lengths of all contours in basin, multiplying by contour interval, and dividing product by drainage area | S |  |  |
|  |  | CONTOUR | | Total length of all elevation contours in drainage area in miles | S |  |  |
|  |  | CSL10_85 | | Change in elevation divided by length between points 10 and 85 percent of distance along main channel to basin divide | S |  |  |
|  |  | CSL1085LO | | Change in elevation between points 10 and 85 percent of length along the lower half of the main flow path divided by length between the points | S |  |  |
|  |  | CSL1085UP | | Change in elevation between points 10 and 85 percent of length along the upper half of the main flow path divided by length between the points | S |  |  |
|  |  | EL1200 | | Percentage of basin at or above 1200 ft elevation | S |  |  |
|  |  | LAGFACTOR | | Basin Lag factor as defined in SIR 2006-5112 | S |  |  |
|  |  | Length | | Length along the main channel from the measuring location extended to the basin divide | S |  |  |
|  |  | SLOPERAT | | Ratio of main channel slope to basin slope | S |  |  |
|  |  | SSURGOA | | Percentage of area of Hydrologic Soils Type A from SSURGO | S |  |  |
|  |  | SSURGOB | | Percentage of area of Hydrologic Soils Type B from SSURGO | S |  |  |
|  |  | Storage | | Percentage of area of storage (lake, ponds, reservoirs, wetlands) | S |  |  |
|  |  | StreamLevel | | Stream level | S |  |  |
|  |  | StreamOrder | | Strahler stream order | S |  |  |
|  |  | ArbolateSu | | An estimate of miles of stream upstream of a flowline | S |  |  |
| Water Quality and Hydrological Conditions at Time of Sample Collection | | | | |  |  |  |
|  | Air Temp. at site | | | Air temperature measured at the sampling site at the time of sample collection (°C) | Pq | - | - |
|  | *E. coli ^d^* | | | Log_10_ *E. coli* concentration in the waterway (MPN/100 mL) | Mq | - | - |
|  | Conductivity | | | Conductivity (Log_10_ uS/cm) | Pq | - | - |
|  | Dissolved oxygen | | | Dissolved oxygen levels (mg/L) | Pq | - | - |
|  | Flow rate | | | Flow rate measured 3-6” below the surface (m/s) | Pq | - | - |
|  | pH | | | pH | Pq | - | - |
|  | Turbidity | | | Turbidity (Log_10_ NTU) | Pq | - | - |
|  | Water Temp. | | | Water temperature (°C) | Pq | - | - |
| Field-Collected Site Data | | | | |  |  |  |
|  | Ditch | | | Did a roadside ditch intersect the stream < 20 m upstream of the sample site? | FC | ~~-~~ | ~~-~~ |
|  | Bottom Substrate: Composition of the stream bottom in the reach 10 m upstream of the sample site. The different categories of substrate were boulder, bedrock, cobble or larger, coarse gravel, fine gravel, sand, clay and organic matter. | | | |  | ~~-~~ | (*16*) |
|  |  | Rocky | | Was the substrate that comprised the majority of the bottom rocky (bedrock, boulder, cobble, or gravel) or not rocky (sand, clay, or organic matter/silt)? | FC | - | - |
|  |  | Sand | | Was sand present along the stream bottom? | FC | - | - |
|  |  | Clay | | Was clay present along the stream bottom? | FC | - | - |
|  |  | Organic Matter | | Was organic matter present along the stream bottom? | FC | - | - |
|  |  | Cobble or Larger | | Were cobble, boulders or bedrock along the stream bottom? | FC | - | - |
|  |  | Fine gravel | | Was fine gravel present along the stream bottom? | FC | - | - |
|  |  | Coarse gravel | | Was coarse gravel present along the stream bottom? | FC | - | - |
| Weather | | | |  |  |  |  |
|  | Avg. Air Temp. | | | Average temperature (°C) either 0-5, 5-10, 10-20 or 20-30 d before sample collection | W | - | [newa.cornell.edu](http://newa.cornell.edu/) |
|  | Avg. Solar Radiation | | | Average solar radiation (MJ/m^2^) either 0-1,1-2, 2-3, 3-4, 4-5, 5-10, 10-20 or 20-30 d before sample collection | W | - | [newa.cornell.edu](http://newa.cornell.edu/) |
|  | Total rainfall | | | Total rainfall (cm) either 0-1,1-2, 2-3, 3-4, 4-5, 5-10, 10-20 or 20-30 d before sample collection | W | - | [newa.cornell.edu](http://newa.cornell.edu/) |

^a^ To assess the relative information gain associated with using different feature types to build the models, two sets of analyses were performed. In the first set, each learner and the full set of features listed here were used to develop full models. In the second set, the features listed here were divided into groups: Mq = microbial, Turb = turbidity, Pq= physicochemical water quality and air temperature collected on site; W= weather from publicly-available databases; S = spatial. Nested models were then built using different combinations of these feature types.

^b^ New York State = NYS

^c^ For each land cover class we calculated the proportion of (i) the total watershed area, (ii) the stream corridor (i.e., area 0-60 m from the stream corridor), (iii) the flood plain (based on shapefile downloaded from NYS Department of Environmental Conservation), and (iv) the area immediately upstream (0-100 m) of the sampling site.

^d^ Limit of detection = LOD; the upper limit of detection for the *E. coli* and total coliforms assay was 2,419.6 MPN/100-mL.

Table S2: Performance measures for each *Listeria monocytogenes* model developed in the current study. The top-ranked models based on the performance measure in the given column are highlighted in blue, while the worst models, models below a performance cut-off for the given measure (e.g., 0.50 for AUC, 1.0 for DOR, 0.0 for Kappa), and models where the given measure could not be calculated (usually due to poor performance) are highlighted in yellow. Note that a higher rank indicates a better performing model.

| **Learner** | | **Features ^a^** | **Resample**  **Method ^b^** | **Probability**  **Threshold ^c^** | **ACC ^d^** | **AUC ^e^** | **DOR ^f^** | **INF ^g^** | **Kappa ^h^** | **MCC ^i^** | **Sensitivity ^j^** | **Specificity ^k^** | **Rank** |
| --- | --- | --- | --- | --- | --- | --- | --- | --- | --- | --- | --- | --- | --- |
|  |  |  |  |  |  |  |  |  |  |  |  |  |  |
| Binary Cut-offs (MPN/100-mL) | | | | |  |  |  |  |  |  |  |  |  |
|  | 126 | Microbial | None | - | 43% | - | 0.8 | -0.07 | -0.07 | -0.05 | 52% | 41% | 89 |
|  | 235 | Microbial | None | - | 56% | - | 1.0 | 0.00 | 0.00 | 0.00 | 41% | 59% | 93 |
|  | 410 | Microbial | None | - | 68% | - | 0.9 | -0.02 | -0.02 | -0.01 | 22% | 76% | 81 |
| Decision Trees | | | |  |  |  |  |  |  |  |  |  |  |
|  | Classification Trees (CART) | | | |  |  |  |  |  |  |  |  |  |
|  |  | Full | None | 1.0 | 85% | 0.47 | - | 0.00 | 0.00 | - | 0% | 100% | 69 |
|  |  | Full | Over | 0.1 | 72% | 0.51 | 1.2 | 0.03 | 0.02 | 0.02 | 22% | 80% | 81 |
|  |  | Full | SMOTE | 0.4 | 76% | 0.51 | 1.7 | 0.08 | 0.08 | 0.07 | 22% | 86% | 111 |
|  | Conditional Inference Tree (cTree) | | | |  |  |  |  |  |  |  |  |  |
|  |  | Full | None | 1.0 | 85% | 0.50 | - | 0.00 | 0.00 | - | 0% | 100% | 88 |
|  |  | Full | Over | 0.2 | 69% | 0.52 | 1.2 | 0.03 | 0.02 | 0.03 | 26% | 77% | 90 |
|  |  | Full | SMOTE | 0.8 | 85% | 0.44 | - | 0.00 | 0.00 | - | 0% | 100% | 61 |
|  |  | Mq | None | 1.0 | 85% | 0.50 | - | 0.00 | 0.00 | - | 0% | 100% | 98 |
|  |  | Mq | Over | 1.0 | 85% | 0.50 | - | 0.00 | 0.00 | - | 0% | 100% | 93 |
|  |  | Mq | SMOTE | 1.0 | 85% | 0.50 | - | 0.00 | 0.00 | - | 0% | 100% | 91 |
|  |  | MqTurb | None | 1.0 | 85% | 0.50 | - | 0.00 | 0.00 | - | 0% | 100% | 98 |
|  |  | MqTurb | Over | 1.0 | 85% | 0.50 | - | 0.00 | 0.00 | - | 0% | 100% | 93 |
|  |  | MqTurb | SMOTE | 0.2 | 58% | 0.53 | 1.2 | 0.05 | 0.03 | 0.04 | 44% | 61% | 106 |
|  |  | MPq | None | 1.0 | 85% | 0.50 | - | 0.00 | 0.00 | - | 0% | 100% | 98 |
|  |  | MPq | Over | 1.0 | 85% | 0.50 | - | 0.00 | 0.00 | - | 0% | 100% | 93 |
|  |  | MPq | SMOTE | 0.2 | 58% | 0.53 | 1.2 | 0.05 | 0.03 | 0.04 | 44% | 61% | 108 |
|  |  | MPqW | None | 1.0 | 85% | 0.50 | - | 0.00 | 0.00 | - | 0% | 100% | 99 |
|  |  | MPqW | Over | 0.2 | 69% | 0.52 | 1.2 | 0.03 | 0.02 | 0.03 | 26% | 77% | 90 |
|  |  | MPqW | SMOTE | 0.4 | 76% | 0.52 | 1.4 | 0.04 | 0.04 | 0.05 | 19% | 86% | 95 |
|  |  | MqW | None | 1.0 | 85% | 0.50 | - | 0.00 | 0.00 | - | 0% | 100% | 99 |
|  |  | MqW | Over | 0.2 | 69% | 0.52 | 1.2 | 0.03 | 0.02 | 0.03 | 26% | 77% | 90 |
|  |  | MqW | SMOTE | 0.6 | 85% | 0.48 | - | 0.00 | 0.00 | - | 0% | 100% | 75 |
|  |  | Pq | None | 1.0 | 85% | 0.50 | - | 0.00 | 0.00 | - | 0% | 100% | 100 |
|  |  | Pq | Over | 1.0 | 85% | 0.50 | - | 0.00 | 0.00 | - | 0% | 100% | 94 |
|  |  | Pq | SMOTE | 0.1 | 29% | 0.50 | 1.1 | 0.02 | 0.01 | 0.01 | 81% | 20% | 74 |
|  |  | PqW | None | 1.0 | 85% | 0.50 | - | 0.00 | 0.00 | - | 0% | 100% | 100 |
|  |  | PqW | Over | 0.2 | 69% | 0.52 | 1.2 | 0.03 | 0.02 | 0.03 | 26% | 77% | 90 |
|  |  | PqW | SMOTE | 0.2 | 76% | 0.52 | 1.4 | 0.04 | 0.04 | 0.05 | 19% | 86% | 95 |
|  |  | S | None | 1.0 | 85% | 0.50 | - | 0.00 | 0.00 | - | 0% | 100% | 97 |
|  |  | S | Over | 1.0 | 85% | 0.50 | - | 0.00 | 0.00 | - | 0% | 100% | 92 |
|  |  | S | SMOTE | 1.0 | 85% | 0.50 | - | 0.00 | 0.00 | - | 0% | 100% | 91 |
|  |  | W | None | 1.0 | 85% | 0.50 | - | 0.00 | 0.00 | - | 0% | 100% | 99 |
|  |  | W | Over | 0.2 | 69% | 0.52 | 1.2 | 0.03 | 0.02 | 0.03 | 26% | 77% | 90 |
|  |  | W | SMOTE | 0.2 | 33% | 0.52 | 1.8 | 0.09 | 0.03 | 0.07 | 85% | 24% | 111 |
|  | Evolutionary Optimal Trees (EvTree) | | | | |  |  |  |  |  |  |  |  |
|  |  | Full | None | 1.0 | 85% | 0.46 | - | 0.00 | 0.00 | - | 0% | 100% | 65 |
|  |  | Full | Over | 1.0 | 77% | 0.48 | 0.7 | -0.03 | -0.04 | -0.03 | 7% | 90% | 23 |
|  |  | Full | SMOTE | 0.2 | 62% | 0.56 | 1.9 | 0.15 | 0.09 | 0.11 | 52% | 63% | 152 |
| Ensemble Learners | | | | |  |  |  |  |  |  |  |  |  |
|  | Conditional Forests (condRF) | | | |  |  |  |  |  |  |  |  |  |
|  |  | Full | None | 0.2 | 85% | 0.44 | - | 0.00 | 0.00 | - | 0% | 100% | 61 |
|  |  | Full | Over | 0.2 | 74% | 0.48 | 1.4 | 0.05 | 0.05 | 0.05 | 22% | 83% | 69 |
|  |  | Full | SMOTE | 1.0 | 85% | 0.49 | - | 0.00 | 0.00 | - | 0% | 100% | 75 |
|  | Node Harvest (nHarvest) | | | | |  |  |  |  |  |  |  |  |
|  |  | Full | None | 0.2 | 83% | 0.42 | 1.4 | 0.01 | 0.02 | 0.02 | 4% | 97% | 26 |
|  |  | Full | Over | 0.1 | 59% | 0.52 | 1.6 | 0.12 | 0.07 | 0.09 | 52% | 60% | 127 |
|  |  | Full | SMOTE | 0.4 | 82% | 0.48 | 2.8 | 0.09 | 0.11 | 0.12 | 15% | 94% | 90 |
|  | Random Forest (RF) | | | |  |  |  |  |  |  |  |  |  |
|  |  | Full | None | 0.1 | 68% | 0.50 | 1.8 | 0.13 | 0.10 | 0.11 | 41% | 73% | 127 |
|  |  | Full | Over | 0.2 | 78% | 0.50 | 1.8 | 0.07 | 0.08 | 0.08 | 19% | 89% | 107 |
|  |  | Full | SMOTE | 0.3 | 82% | 0.48 | 2.3 | 0.06 | 0.08 | 0.09 | 11% | 95% | 70 |
|  |  | Mq | None | 0.1 | 78% | 0.56 | 2.5 | 0.14 | 0.14 | 0.14 | 26% | 88% | 156 |
|  |  | Mq | Over | 0.4 | 74% | 0.55 | 1.9 | 0.11 | 0.10 | 0.10 | 30% | 82% | 140 |
|  |  | Mq | SMOTE | 0.6 | 83% | 0.56 | 4.1 | 0.16 | 0.19 | 0.19 | 22% | 93% | 164 |
|  |  | MqTurb | None | 0.2 | 83% | 0.45 | 1.1 | 0.00 | 0.01 | 0.01 | 4% | 97% | 25 |
|  |  | MqTurb | Over | 0.0 | 36% | 0.47 | 2.8 | 0.15 | 0.06 | 0.13 | 89% | 26% | 89 |
|  |  | MqTurb | SMOTE | 0.2 | 67% | 0.52 | 2.0 | 0.16 | 0.11 | 0.12 | 44% | 71% | 145 |
|  |  | MPq | None | 0.1 | 76% | 0.58 | 2.3 | 0.14 | 0.13 | 0.13 | 30% | 84% | 159 |
|  |  | MPq | Over | 0.1 | 33% | 0.55 | 7.2 | 0.18 | 0.06 | 0.16 | 96% | 22% | 144 |
|  |  | MPq | SMOTE | 0.5 | 85% | 0.50 | 5.9 | 0.03 | 0.05 | 0.10 | 4% | 99% | 72 |
|  |  | MPqW | None | 0.2 | 81% | 0.47 | 2.0 | 0.07 | 0.08 | 0.09 | 15% | 92% | 70 |
|  |  | MPqW | Over | 0.2 | 81% | 0.56 | 2.7 | 0.11 | 0.13 | 0.13 | 19% | 92% | 145 |
|  |  | MPqW | SMOTE | 0.4 | 85% | 0.53 | 6.3 | 0.09 | 0.13 | 0.18 | 11% | 98% | 132 |
|  |  | MqW | None | 0.0 | 26% | 0.47 | 3.9 | 0.09 | 0.03 | 0.10 | 96% | 13% | 71 |
|  |  | MqW | Over | 0.2 | 80% | 0.52 | 2.3 | 0.09 | 0.11 | 0.11 | 19% | 91% | 126 |
|  |  | MqW | SMOTE | 0.4 | 82% | 0.56 | 2.8 | 0.09 | 0.11 | 0.12 | 15% | 94% | 137 |
|  |  | Pq | None | 0.1 | 78% | 0.60 | 3.3 | 0.22 | 0.20 | 0.20 | 37% | 85% | 176 |
|  |  | Pq | Over | 0.1 | 71% | 0.58 | 1.9 | 0.14 | 0.10 | 0.11 | 37% | 76% | 154 |
|  |  | Pq | SMOTE | 0.7 | 86% | 0.52 | - | 0.04 | 0.06 | 0.18 | 4% | 100% | 90 |
|  |  | PqW | None | 0.1 | 81% | 0.48 | 2.0 | 0.07 | 0.08 | 0.09 | 15% | 92% | 77 |
|  |  | PqW | Over | 0.2 | 81% | 0.55 | 2.3 | 0.08 | 0.09 | 0.10 | 15% | 93% | 126 |
|  |  | PqW | SMOTE | 0.5 | 86% | 0.55 | - | 0.07 | 0.12 | 0.25 | 7% | 100% | 129 |
|  |  | S | None | 0.0 | 79% | 0.50 | 2.5 | 0.12 | 0.13 | 0.13 | 22% | 90% | 110 |
|  |  | S | Over | 0.1 | 79% | 0.49 | 2.5 | 0.12 | 0.13 | 0.13 | 22% | 90% | 104 |
|  |  | S | SMOTE | 0.1 | 79% | 0.50 | 2.5 | 0.12 | 0.13 | 0.13 | 22% | 90% | 109 |
|  |  | W | None | 0.0 | 31% | 0.47 | 2.0 | 0.09 | 0.03 | 0.08 | 89% | 20% | 71 |
|  |  | W | Over | 0.2 | 78% | 0.52 | 1.7 | 0.07 | 0.07 | 0.07 | 19% | 88% | 114 |
|  |  | W | SMOTE | 0.4 | 84% | 0.60 | 5.2 | 0.12 | 0.16 | 0.19 | 15% | 97% | 154 |
|  | Regularized Random Forest (regRF) | | | | |  |  |  |  |  |  |  |  |
|  |  | Full | None | 0.6 | 85% | 0.34 | - | 0.00 | 0.00 | - | 0% | 100% | 63 |
|  |  | Full | Over | 0.1 | 68% | 0.49 | 1.3 | 0.05 | 0.04 | 0.04 | 30% | 75% | 70 |
|  |  | Full | SMOTE | 0.7 | 84% | 0.48 | 2.9 | 0.02 | 0.04 | 0.07 | 4% | 99% | 44 |
| Instance-Based | | |  |  |  |  |  |  |  |  |  |  |  |
|  | K-Nearest Neighbor (kKNN) | | |  |  |  |  |  |  |  |  |  |  |
|  |  | Full | None | 0.2 | 83% | 0.54 | 4.1 | 0.13 | 0.17 | 0.19 | 19% | 95% | 149 |
|  |  | Full | Over | 1.0 | 81% | 0.49 | 0.7 | -0.02 | -0.02 | -0.02 | 4% | 95% | 18 |
|  |  | Full | SMOTE | 0.2 | 79% | 0.53 | 1.7 | 0.06 | 0.07 | 0.07 | 15% | 91% | 111 |
|  | Weighted K-Nearest Neighbor (wKNN) | | | |  |  |  |  |  |  |  |  |  |
|  |  | Full | None | 0.4 | 82% | 0.52 | 2.8 | 0.09 | 0.11 | 0.12 | 15% | 94% | 121 |
|  |  | Full | Over | 0.1 | 29% | 0.50 | - | 0.16 | 0.06 | 0.16 | 100% | 16% | 117 |
|  |  | Full | SMOTE | 0.4 | 82% | 0.52 | 2.8 | 0.09 | 0.11 | 0.12 | 15% | 94% | 121 |
| Penalized Regression | | | | |  |  |  |  |  |  |  |  |  |
|  | Lasso | | |  |  |  |  |  |  |  |  |  |  |
|  |  | Full | None | 1.0 | 85% | 0.50 | - | 0.00 | 0.00 | - | 0% | 100% | 89 |
|  |  | Full | Over | 0.9 | 86% | 0.52 | 12.2 | 0.07 | 0.11 | 0.18 | 7% | 99% | 114 |
|  |  | Full | SMOTE | 0.7 | 83% | 0.51 | 3.2 | 0.10 | 0.12 | 0.14 | 15% | 95% | 120 |
|  | Ridge | | |  |  |  |  |  |  |  |  |  |  |
|  |  | Full | None | 1.0 | 85% | 0.50 | - | 0.00 | 0.00 | - | 0% | 100% | 90 |
|  |  | Full | Over | 0.1 | 71% | 0.55 | 1.9 | 0.14 | 0.10 | 0.11 | 37% | 76% | 149 |
|  |  | Full | SMOTE | 0.8 | 86% | 0.53 | - | 0.04 | 0.06 | 0.18 | 4% | 100% | 99 |
|  |  | MqTurb | None | 1.0 | 85% | 0.50 | - | 0.00 | 0.00 | - | 0% | 100% | 95 |
|  |  | MqTurb | Over | 0.4 | 77% | 0.52 | 2.1 | 0.12 | 0.11 | 0.11 | 26% | 86% | 136 |
|  |  | MqTurb | SMOTE | 0.3 | 71% | 0.50 | 1.8 | 0.11 | 0.09 | 0.09 | 33% | 78% | 103 |
|  |  | MPq | None | 1.0 | 85% | 0.50 | - | 0.00 | 0.00 | - | 0% | 100% | 96 |
|  |  | MPq | Over | 0.4 | 75% | 0.49 | 2.1 | 0.13 | 0.11 | 0.12 | 30% | 83% | 108 |
|  |  | MPq | SMOTE | 0.5 | 83% | 0.50 | 2.6 | 0.07 | 0.09 | 0.10 | 11% | 95% | 101 |
|  |  | MPqW | None | 1.0 | 85% | 0.50 | - | 0.00 | 0.00 | - | 0% | 100% | 96 |
|  |  | MPqW | Over | 1.0 | 86% | 0.50 | - | 0.04 | 0.06 | 0.18 | 4% | 100% | 81 |
|  |  | MPqW | SMOTE | 1.0 | 86% | 0.47 | - | 0.04 | 0.06 | 0.18 | 4% | 100% | 52 |
|  |  | MqW | None | 1.0 | 85% | 0.50 | - | 0.00 | 0.00 | - | 0% | 100% | 96 |
|  |  | MqW | Over | 0.2 | 58% | 0.56 | 1.6 | 0.11 | 0.06 | 0.08 | 52% | 59% | 137 |
|  |  | MqW | SMOTE | 0.9 | 86% | 0.48 | - | 0.04 | 0.06 | 0.18 | 4% | 100% | 56 |
|  |  | Pq | None | 1.0 | 85% | 0.50 | - | 0.00 | 0.00 | - | 0% | 100% | 97 |
|  |  | Pq | Over | 0.4 | 74% | 0.48 | 2.0 | 0.12 | 0.11 | 0.11 | 30% | 82% | 97 |
|  |  | Pq | SMOTE | 0.4 | 73% | 0.47 | 1.5 | 0.07 | 0.06 | 0.06 | 26% | 81% | 69 |
|  |  | PqW | None | 1.0 | 85% | 0.50 | - | 0.00 | 0.00 | - | 0% | 100% | 95 |
|  |  | PqW | Over | 1.0 | 86% | 0.49 | - | 0.04 | 0.06 | 0.18 | 4% | 100% | 63 |
|  |  | PqW | SMOTE | 0.9 | 86% | 0.49 | - | 0.04 | 0.06 | 0.18 | 4% | 100% | 62 |
|  |  | S | None | 1.0 | 85% | 0.50 | - | 0.00 | 0.00 | - | 0% | 100% | 95 |
|  |  | S | Over | 0.2 | 86% | 0.50 | - | 0.04 | 0.06 | 0.18 | 4% | 100% | 65 |
|  |  | S | SMOTE | 0.1 | 31% | 0.50 | 3.1 | 0.12 | 0.04 | 0.12 | 93% | 20% | 94 |
|  |  | W | None | 1.0 | 85% | 0.50 | - | 0.00 | 0.00 | - | 0% | 100% | 97 |
|  |  | W | Over | 0.2 | 47% | 0.55 | 2.5 | 0.20 | 0.09 | 0.14 | 78% | 42% | 151 |
|  |  | W | SMOTE | 0.6 | 86% | 0.46 | - | 0.04 | 0.06 | 0.18 | 4% | 100% | 49 |
| Rule-Based | | | |  |  |  |  |  |  |  |  |  |  |
|  | JRip | | |  |  |  |  |  |  |  |  |  |  |
|  |  | Full | None | 1.0 | 83% | 0.49 | 0.0 | -0.03 | -0.04 | -0.07 | 0% | 97% | 65 |
|  |  | Full | Over | 1.0 | 76% | 0.49 | 0.9 | -0.01 | -0.01 | -0.01 | 11% | 88% | 31 |
|  |  | Full | SMOTE | 0.0 | 15% | 0.47 | - | 0.00 | 0.00 | - | 100% | 0% | 52 |
|  | Partial Decision Trees (PART) | | | |  |  |  |  |  |  |  |  |  |
|  |  | Full | None | 0.1 | 32% | 0.51 | 1.1 | 0.01 | 0.00 | 0.01 | 78% | 24% | 91 |
|  |  | Full | Over | 0.9 | 83% | 0.49 | 1.1 | 0.00 | 0.01 | 0.01 | 4% | 97% | 42 |
|  |  | Full | SMOTE | 0.9 | 86% | 0.54 | 12.2 | 0.07 | 0.11 | 0.18 | 7% | 99% | 121 |
|  |  | Mq | None | 1.0 | 90% | 0.50 | - | 0.00 | 0.00 | - | 0% | 100% | 102 |
|  |  | Mq | Over | 1.0 | 70% | 0.50 | - | 0.00 | 0.00 | - | 0% | 100% | 94 |
|  |  | Mq | SMOTE | 1.0 | 70% | 0.50 | - | 0.00 | 0.00 | - | 0% | 100% | 92 |
|  |  | MqTurb | None | 1.0 | 85% | 0.50 | - | 0.00 | 0.00 | - | 0% | 100% | 102 |
|  |  | MqTurb | Over | 0.4 | 80% | 0.58 | 2.3 | 0.09 | 0.11 | 0.11 | 19% | 91% | 143 |
|  |  | MqTurb | SMOTE | 0.8 | 85% | 0.52 | 5.9 | 0.03 | 0.05 | 0.10 | 4% | 99% | 84 |
|  |  | MPq | None | 1.0 | 85% | 0.50 | - | 0.00 | 0.00 | - | 0% | 100% | 102 |
|  |  | MPq | Over | 1.0 | 83% | 0.46 | 1.4 | 0.01 | 0.02 | 0.02 | 4% | 97% | 29 |
|  |  | MPq | SMOTE | 0.1 | 43% | 0.53 | 1.5 | 0.08 | 0.04 | 0.06 | 70% | 38% | 117 |
|  |  | MPqW | None | 0.0 | 28% | 0.53 | 4.8 | 0.12 | 0.04 | 0.12 | 96% | 16% | 127 |
|  |  | MPqW | Over | 0.9 | 82% | 0.50 | 1.7 | 0.03 | 0.04 | 0.04 | 7% | 95% | 76 |
|  |  | MPqW | SMOTE | 0.8 | 75% | 0.57 | 2.3 | 0.16 | 0.14 | 0.14 | 33% | 82% | 165 |
|  |  | MqW | None | 1.0 | 85% | 0.50 | - | 0.00 | 0.00 | - | 0% | 100% | 103 |
|  |  | MqW | Over | 0.6 | 81% | 0.46 | 1.1 | 0.01 | 0.01 | 0.01 | 7% | 93% | 35 |
|  |  | MqW | SMOTE | 0.8 | 79% | 0.53 | 2.8 | 0.15 | 0.16 | 0.15 | 26% | 89% | 150 |
|  |  | Pq | None | 1.0 | 85% | 0.50 | - | 0.00 | 0.00 | - | 0% | 100% | 103 |
|  |  | Pq | Over | 1.0 | 83% | 0.46 | 2.4 | 0.04 | 0.06 | 0.08 | 7% | 97% | 51 |
|  |  | Pq | SMOTE | 0.1 | 34% | 0.52 | 1.6 | 0.08 | 0.03 | 0.06 | 81% | 26% | 108 |
|  |  | PqW | None | 0.0 | 28% | 0.53 | 4.8 | 0.12 | 0.04 | 0.12 | 96% | 16% | 127 |
|  |  | PqW | Over | 0.9 | 82% | 0.50 | 1.7 | 0.03 | 0.04 | 0.04 | 7% | 95% | 76 |
|  |  | PqW | SMOTE | 0.4 | 63% | 0.56 | 2.0 | 0.17 | 0.10 | 0.12 | 52% | 65% | 158 |
|  |  | S | None | 1.0 | 85% | 0.50 | - | 0.00 | 0.00 | - | 0% | 100% | 101 |
|  |  | S | Over | 1.0 | 85% | 0.49 | - | 0.00 | 0.00 | - | 0% | 100% | 84 |
|  |  | S | SMOTE | 0.1 | 72% | 0.47 | 1.4 | 0.06 | 0.05 | 0.05 | 26% | 80% | 66 |
|  |  | W | None | 1.0 | 85% | 0.45 | - | 0.00 | 0.00 | - | 0% | 100% | 78 |
|  |  | W | Over | 0.6 | 81% | 0.46 | 1.1 | 0.01 | 0.01 | 0.01 | 7% | 93% | 35 |
|  |  | W | SMOTE | 0.1 | 64% | 0.53 | 1.3 | 0.06 | 0.04 | 0.05 | 37% | 69% | 114 |
| Support Vector Machines (SVM) | | | | | | |  |  |  |  |  |  |  |
|  | Linear Hyperplane (SVM Linear) | | |  |  |  |  |  |  |  |  |  |  |
|  |  | Full | None | 0.1 | 46% | 0.53 | 2.0 | 0.15 | 0.06 | 0.11 | 74% | 41% | 139 |
|  |  | Full | Over | 0.4 | 84% | 0.52 | 4.7 | 0.08 | 0.12 | 0.15 | 11% | 97% | 118 |
|  |  | Full | SMOTE | 0.3 | 68% | 0.58 | 2.4 | 0.20 | 0.14 | 0.15 | 48% | 72% | 172 |
|  |  | Mq | None | 0.2 | 70% | 0.50 | 1.4 | 0.07 | 0.05 | 0.06 | 30% | 77% | 84 |
|  |  | Mq | Over | 0.3 | 28% | 0.50 | 2.4 | 0.09 | 0.03 | 0.09 | 93% | 16% | 98 |
|  |  | Mq | SMOTE | 0.3 | 29% | 0.50 | 2.7 | 0.10 | 0.04 | 0.10 | 93% | 18% | 106 |
|  |  | MqTurb | None | 0.1 | 77% | 0.44 | 1.6 | 0.06 | 0.06 | 0.07 | 19% | 88% | 63 |
|  |  | MqTurb | Over | 0.3 | 83% | 0.57 | 4.5 | 0.19 | 0.22 | 0.23 | 26% | 93% | 173 |
|  |  | MqTurb | SMOTE | 0.4 | 84% | 0.52 | 3.7 | 0.08 | 0.11 | 0.14 | 11% | 97% | 117 |
|  |  | MPq | None | 0.1 | 31% | 0.49 | 3.2 | 0.13 | 0.05 | 0.12 | 93% | 20% | 92 |
|  |  | MPq | Over | 0.5 | 83% | 0.49 | 2.4 | 0.04 | 0.06 | 0.08 | 7% | 97% | 61 |
|  |  | MPq | SMOTE | 0.3 | 72% | 0.51 | 1.6 | 0.09 | 0.07 | 0.07 | 30% | 79% | 114 |
|  |  | MPqW | None | 1.0 | 85% | 0.47 | - | 0.00 | 0.00 | - | 0% | 100% | 79 |
|  |  | MPqW | Over | 0.9 | 86% | 0.48 | - | 0.04 | 0.06 | 0.18 | 4% | 100% | 58 |
|  |  | MPqW | SMOTE | 1.0 | 86% | 0.49 | - | 0.04 | 0.06 | 0.18 | 4% | 100% | 63 |
|  |  | MqW | None | 0.1 | 16% | 0.53 | - | 0.01 | 0.00 | 0.04 | 100% | 1% | 105 |
|  |  | MqW | Over | 0.4 | 79% | 0.55 | 1.7 | 0.06 | 0.07 | 0.07 | 15% | 91% | 116 |
|  |  | MqW | SMOTE | 0.9 | 86% | 0.48 | - | 0.04 | 0.06 | 0.18 | 4% | 100% | 55 |
|  |  | Pq | None | 0.1 | 83% | 0.51 | 4.1 | 0.16 | 0.19 | 0.19 | 22% | 93% | 144 |
|  |  | Pq | Over | 0.4 | 83% | 0.48 | 3.1 | 0.07 | 0.10 | 0.12 | 11% | 96% | 78 |
|  |  | Pq | SMOTE | 1.0 | 85% | 0.48 | - | 0.00 | 0.00 | - | 0% | 100% | 74 |
|  |  | PqW | None | 1.0 | 85% | 0.48 | - | 0.00 | 0.00 | - | 0% | 100% | 81 |
|  |  | PqW | Over | 0.9 | 86% | 0.48 | - | 0.04 | 0.06 | 0.18 | 4% | 100% | 56 |
|  |  | PqW | SMOTE | 1.0 | 86% | 0.50 | - | 0.04 | 0.06 | 0.18 | 4% | 100% | 66 |
|  |  | S | None | 0.1 | 31% | 0.54 | 3.1 | 0.12 | 0.04 | 0.12 | 93% | 20% | 129 |
|  |  | S | Over | 0.1 | 31% | 0.48 | 1.2 | 0.03 | 0.01 | 0.02 | 81% | 22% | 66 |
|  |  | S | SMOTE | 0.2 | 44% | 0.56 | 1.8 | 0.13 | 0.05 | 0.10 | 74% | 39% | 139 |
|  |  | W | None | 1.0 | 85% | 0.53 | - | 0.00 | 0.00 | - | 0% | 100% | 123 |
|  |  | W | Over | 0.4 | 78% | 0.52 | 2.0 | 0.10 | 0.10 | 0.10 | 22% | 88% | 124 |
|  |  | W | SMOTE | 0.2 | 53% | 0.54 | 2.4 | 0.21 | 0.10 | 0.15 | 70% | 50% | 154 |
|  | Polynomial Hyperplane (SVM Polynomial) | | | |  |  |  |  |  |  |  |  |  |
|  |  | Full | None | 0.1 | 33% | 0.47 | 1.8 | 0.09 | 0.03 | 0.07 | 85% | 24% | 72 |
|  |  | Full | Over | 0.1 | 82% | 0.52 | 2.3 | 0.06 | 0.08 | 0.09 | 11% | 95% | 107 |
|  |  | Full | SMOTE | 0.2 | 84% | 0.51 | 3.0 | 0.05 | 0.07 | 0.09 | 7% | 97% | 94 |

^a^ To assess the relative information gain associated with using different feature types to build the models, two sets of analyses were performed. In the first set, each learner and the full set of features (Table S1) were used to develop full models. In the second set, the features listed in Table S1 were divided into four groups: Mq = microbial, Turb = turbidity, Pq= physicochemical water quality and air temperature collected on site; W= weather from publicly-available databases; S = spatial. Nested models were then built using different combinations of these feature types.

^b^ To assess the effect of resampling on model performance, two resampling methods [oversampling (over) and synthetic minority oversampling (SMOTE)] as well as no resampling (None) were used to develop three, separate models for each outcome-learner-feature type combination (e.g., *L. monocytogenes*-Ridge regressionFull Model).

^c^ After model tuning and training, predictive performance was assessed using the test data. The probability threshold was tuned to maximize kappa score, since the values of several performance measures (e.g., sensitivity) are dependent on this threshold.

^d^ ACC = Accuracy.

^e^ AUC = Area under the Curve. AUC equals the probability that the model will rank a randomly chosen positive sample higher than a randomly chosen negative sample. AUC ranges between 0 and 1, with AUC = 1.0 indicating perfect prediction. AUC ≤ 0.5 indicates that the model’s performance is equal to or worse than chance.

^f^ DOR = Diagnostic Odds Ratio. DOR ≤ 1 indicates a poor performing models (i.e., a model where a positive prediction is associated with a sample testing negative for *L. monocytogenes*). DOR > 1 indicates that a positive prediction is associated with the sample testing positive for *L. monocytogenes* [see (*2*)].

^g^ INF = Informedness, a measure of how informed the model is about both positive and negative samples. A value of 1 means that the pathogen status of all samples was correctly predicted, while a value of -1 implies the incorrect pathogen-status was predicted for all samples [see (*1*)].

^h^ Kappa score represents how much better the model performs compared to a model that randomly calls novel samples pathogen positive or negative. A score ≤ 0.0 indicates that the model is essentially useless, while a score = 1 indicates a model that always correctly identifies novel samples.

^i^ Matthew’s Correlation Coefficient. MCC ranges between -1 (the model always incorrectly predicts pathogen status) and 1 (the model always correctly predicts pathogen status). MCC ≤ 0.0 indicates that the model is equal to or worse than a model that randomly predicts pathogen status.

^j^ Sensitivity or true positive rate.

^k^ Specificity or true negative rate.

Table S3: Performance measures for each nonpathogenic *Listeria* spp. (excluding *L. monocytogenes*) model developed in the current study. The top-ranked models based on the performance measure in the given column are highlighted in blue, while the worst models, models below a performance cut-off for the given measure (e.g., 0.50 for AUC, 1.0 for DOR, 0.0 for Kappa), and models where the given measure could not be calculated (usually due to poor performance) are highlighted in yellow. Note that a higher rank indicates a better performing model.

| **Learner** | | **Features ^a^** | **Resample**  **Method ^b^** | **Probability**  **Threshold ^c^** | **ACC ^d^** | **AUC ^e^** | **DOR ^f^** | **INF ^g^** | **Kappa**  **^h^** | **MCC ^i^** | **Sensitivity ^j^** | **Specificity ^k^** | **Rank** |
| --- | --- | --- | --- | --- | --- | --- | --- | --- | --- | --- | --- | --- | --- |
|  |  |  |  |  |  |  |  |  |  |  |  |  |  |
| Binary Cut-offs (MPN/100-mL) | | | | |  |  |  |  |  |  |  |  |  |
|  | 126 | Mq | None | - | 43% | - | 0.6 | -0.11 | -0.11 | -0.10 | 50% | 39% | 66 |
|  | 235 | Mq | None | - | 49% | - | 0.7 | -0.10 | -0.10 | -0.09 | 34% | 56% | 67 |
|  | 410 | Mq | None | - | 52% | - | 0.3 | -0.19 | -0.20 | -0.20 | 11% | 70% | 65 |
| Decision Trees | | | |  |  |  |  |  |  |  |  |  |  |
|  | Classification Trees (CART) | | | |  |  |  |  |  |  |  |  |  |
|  |  | Full | None | 0.6 | 72% | 0.70 | 4.6 | 0.31 | 0.33 | 0.32 | 48% | 83% | 150 |
|  |  | Full | Over | 0.6 | 72% | 0.66 | 4.6 | 0.31 | 0.33 | 0.32 | 48% | 83% | 138 |
|  |  | Full | SMOTE | 0.4 | 71% | 0.70 | 4.4 | 0.34 | 0.33 | 0.33 | 57% | 77% | 160 |
|  | Conditional Inference Tree (cTree) | | | |  |  |  |  |  |  |  |  |  |
|  |  | Full | None | 0.2 | 58% | 0.65 | 4.5 | 0.30 | 0.24 | 0.29 | 84% | 46% | 119 |
|  |  | Full | Over | 0.2 | 58% | 0.65 | 4.5 | 0.30 | 0.24 | 0.29 | 84% | 46% | 119 |
|  |  | Full | SMOTE | 0.2 | 58% | 0.65 | 4.5 | 0.30 | 0.24 | 0.29 | 84% | 46% | 119 |
|  |  | Mq | None | 0.2 | 61% | 0.57 | 1.8 | 0.14 | 0.13 | 0.13 | 46% | 67% | 40 |
|  |  | Mq | Over | 0.2 | 61% | 0.57 | 1.8 | 0.14 | 0.13 | 0.13 | 46% | 67% | 40 |
|  |  | Mq | SMOTE | 0.2 | 61% | 0.57 | 1.8 | 0.14 | 0.13 | 0.13 | 46% | 67% | 40 |
|  |  | MqTurb | None | 1.0 | 69% | 0.50 | - | 0.00 | 0.00 | - | 0% | 100% | 74 |
|  |  | MqTurb | Over | 1.0 | 69% | 0.50 | - | 0.00 | 0.00 | - | 0% | 100% | 71 |
|  |  | MqTurb | SMOTE | 1.0 | 69% | 0.50 | - | 0.00 | 0.00 | - | 0% | 100% | 68 |
|  |  | MPq | None | 1.0 | 69% | 0.50 | - | 0.00 | 0.00 | - | 0% | 100% | 75 |
|  |  | MPq | Over | 1.0 | 69% | 0.50 | - | 0.00 | 0.00 | - | 0% | 100% | 72 |
|  |  | MPq | SMOTE | 1.0 | 69% | 0.50 | - | 0.00 | 0.00 | - | 0% | 100% | 68 |
|  |  | MPqW | None | 1.0 | 69% | 0.50 | - | 0.00 | 0.00 | - | 0% | 100% | 75 |
|  |  | MPqW | Over | 1.0 | 69% | 0.50 | - | 0.00 | 0.00 | - | 0% | 100% | 72 |
|  |  | MPqW | SMOTE | 1.0 | 69% | 0.50 | - | 0.00 | 0.00 | - | 0% | 100% | 68 |
|  |  | MqW | None | 1.0 | 69% | 0.50 | - | 0.00 | 0.00 | - | 0% | 100% | 75 |
|  |  | MqW | Over | 1.0 | 69% | 0.50 | - | 0.00 | 0.00 | - | 0% | 100% | 72 |
|  |  | MqW | SMOTE | 1.0 | 69% | 0.50 | - | 0.00 | 0.00 | - | 0% | 100% | 69 |
|  |  | Pq | None | 1.0 | 69% | 0.50 | - | 0.00 | 0.00 | - | 0% | 100% | 76 |
|  |  | Pq | Over | 1.0 | 69% | 0.50 | - | 0.00 | 0.00 | - | 0% | 100% | 73 |
|  |  | Pq | SMOTE | 0.4 | 72% | 0.67 | 4.1 | 0.27 | 0.29 | 0.29 | 41% | 86% | 109 |
|  |  | PqW | None | 1.0 | 69% | 0.50 | - | 0.00 | 0.00 | - | 0% | 100% | 76 |
|  |  | PqW | Over | 1.0 | 69% | 0.50 | - | 0.00 | 0.00 | - | 0% | 100% | 73 |
|  |  | PqW | SMOTE | 1.0 | 69% | 0.50 | - | 0.00 | 0.00 | - | 0% | 100% | 69 |
|  |  | S | None | 0.2 | 58% | 0.65 | 4.5 | 0.30 | 0.24 | 0.29 | 84% | 46% | 119 |
|  |  | S | Over | 0.2 | 58% | 0.65 | 4.5 | 0.30 | 0.24 | 0.29 | 84% | 46% | 119 |
|  |  | S | SMOTE | 0.2 | 58% | 0.65 | 4.5 | 0.30 | 0.24 | 0.29 | 84% | 46% | 119 |
|  |  | W | None | 1.0 | 69% | 0.50 | - | 0.00 | 0.00 | - | 0% | 100% | 76 |
|  |  | W | Over | 1.0 | 69% | 0.50 | - | 0.00 | 0.00 | - | 0% | 100% | 73 |
|  |  | W | SMOTE | 1.0 | 69% | 0.50 | - | 0.00 | 0.00 | - | 0% | 100% | 69 |
|  | Evolutionary Optimal Trees (EvTree) | | | | |  |  |  |  |  |  |  |  |
|  |  | Full | None | 0.8 | 73% | 0.63 | 5.1 | 0.29 | 0.32 | 0.33 | 41% | 88% | 108 |
|  |  | Full | Over | 0.4 | 67% | 0.69 | 4.2 | 0.34 | 0.31 | 0.32 | 68% | 66% | 150 |
|  |  | Full | SMOTE | 0.6 | 74% | 0.62 | 5.5 | 0.32 | 0.34 | 0.35 | 45% | 87% | 126 |
| Ensemble Learners | | | | |  |  |  |  |  |  |  |  |  |
|  | Conditional Forests (condRF) | | | |  |  |  |  |  |  |  |  |  |
|  |  | Full | None | 0.4 | 71% | 0.73 | 5.4 | 0.40 | 0.37 | 0.37 | 68% | 72% | 180 |
|  |  | Full | Over | 0.4 | 71% | 0.73 | 5.4 | 0.40 | 0.37 | 0.37 | 68% | 72% | 180 |
|  |  | Full | SMOTE | 0.4 | 73% | 0.73 | 6.1 | 0.41 | 0.40 | 0.39 | 62% | 78% | 181 |
|  | Node Harvest (nHarvest) | | | | |  |  |  |  |  |  |  |  |
|  |  | Full | None | 0.6 | 73% | 0.69 | 5.1 | 0.31 | 0.33 | 0.34 | 45% | 86% | 144 |
|  |  | Full | Over | 0.4 | 74% | 0.72 | 6.0 | 0.40 | 0.40 | 0.39 | 59% | 81% | 179 |
|  |  | Full | SMOTE | 0.7 | 75% | 0.66 | 6.2 | 0.29 | 0.33 | 0.35 | 38% | 91% | 119 |
|  | Random Forest (RF) | | | |  |  |  |  |  |  |  |  |  |
|  |  | Full | None | 0.4 | 72% | 0.70 | 4.7 | 0.32 | 0.33 | 0.33 | 50% | 82% | 157 |
|  |  | Full | Over | 0.5 | 73% | 0.71 | 5.2 | 0.33 | 0.35 | 0.35 | 48% | 85% | 165 |
|  |  | Full | SMOTE | 0.5 | 75% | 0.70 | 6.1 | 0.30 | 0.33 | 0.35 | 39% | 90% | 140 |
|  |  | Mq | None | 0.3 | 64% | 0.60 | 2.2 | 0.18 | 0.18 | 0.18 | 46% | 72% | 59 |
|  |  | Mq | Over | 0.2 | 59% | 0.59 | 2.0 | 0.17 | 0.15 | 0.16 | 59% | 58% | 55 |
|  |  | Mq | SMOTE | 0.4 | 69% | 0.60 | 2.7 | 0.16 | 0.18 | 0.19 | 29% | 87% | 46 |
|  |  | MqTurb | None | 0.5 | 74% | 0.63 | 8.0 | 0.21 | 0.26 | 0.32 | 25% | 96% | 68 |
|  |  | MqTurb | Over | 0.6 | 72% | 0.60 | 4.9 | 0.19 | 0.22 | 0.27 | 25% | 94% | 51 |
|  |  | MqTurb | SMOTE | 0.3 | 66% | 0.63 | 3.2 | 0.28 | 0.26 | 0.26 | 57% | 70% | 99 |
|  |  | MPq | None | 0.5 | 71% | 0.63 | 3.8 | 0.22 | 0.25 | 0.26 | 34% | 88% | 72 |
|  |  | MPq | Over | 0.5 | 72% | 0.63 | 4.1 | 0.23 | 0.26 | 0.28 | 34% | 89% | 77 |
|  |  | MPq | SMOTE | 0.5 | 72% | 0.65 | 4.5 | 0.23 | 0.26 | 0.28 | 32% | 90% | 82 |
|  |  | MPqW | None | 0.4 | 70% | 0.64 | 3.3 | 0.23 | 0.25 | 0.25 | 41% | 82% | 83 |
|  |  | MPqW | Over | 0.4 | 72% | 0.65 | 4.1 | 0.25 | 0.27 | 0.29 | 38% | 87% | 91 |
|  |  | MPqW | SMOTE | 0.4 | 70% | 0.65 | 3.3 | 0.21 | 0.23 | 0.25 | 36% | 86% | 82 |
|  |  | MqW | None | 0.5 | 73% | 0.56 | 5.2 | 0.26 | 0.30 | 0.31 | 36% | 90% | 72 |
|  |  | MqW | Over | 0.5 | 73% | 0.57 | 5.2 | 0.26 | 0.30 | 0.31 | 36% | 90% | 75 |
|  |  | MqW | SMOTE | 0.5 | 71% | 0.57 | 3.6 | 0.23 | 0.25 | 0.26 | 38% | 86% | 64 |
|  |  | Pq | None | 0.4 | 67% | 0.63 | 2.7 | 0.21 | 0.22 | 0.22 | 45% | 77% | 70 |
|  |  | Pq | Over | 0.5 | 70% | 0.63 | 3.3 | 0.20 | 0.23 | 0.23 | 34% | 86% | 64 |
|  |  | Pq | SMOTE | 0.5 | 70% | 0.65 | 3.4 | 0.22 | 0.24 | 0.25 | 38% | 85% | 82 |
|  |  | PqW | None | 0.3 | 66% | 0.65 | 3.4 | 0.30 | 0.27 | 0.27 | 62% | 67% | 122 |
|  |  | PqW | Over | 0.4 | 73% | 0.65 | 5.2 | 0.26 | 0.30 | 0.31 | 36% | 90% | 96 |
|  |  | PqW | SMOTE | 0.5 | 74% | 0.65 | 8.0 | 0.21 | 0.26 | 0.32 | 25% | 96% | 81 |
|  |  | S | None | 0.4 | 72% | 0.70 | 4.6 | 0.31 | 0.33 | 0.32 | 48% | 83% | 148 |
|  |  | S | Over | 0.4 | 72% | 0.70 | 4.6 | 0.31 | 0.33 | 0.32 | 48% | 83% | 148 |
|  |  | S | SMOTE | 0.4 | 72% | 0.70 | 4.6 | 0.31 | 0.33 | 0.32 | 48% | 83% | 148 |
|  |  | W | None | 0.4 | 62% | 0.54 | 1.6 | 0.09 | 0.09 | 0.10 | 36% | 74% | 26 |
|  |  | W | Over | 0.4 | 67% | 0.57 | 2.4 | 0.16 | 0.18 | 0.18 | 34% | 82% | 42 |
|  |  | W | SMOTE | 0.4 | 64% | 0.56 | 1.9 | 0.14 | 0.14 | 0.13 | 39% | 74% | 37 |
|  | Regularized Random Forest (regRF) | | | | |  |  |  |  |  |  |  |  |
|  |  | Full | None | 0.6 | 73% | 0.72 | 5.0 | 0.33 | 0.34 | 0.34 | 50% | 83% | 170 |
|  |  | Full | Over | 0.6 | 75% | 0.70 | 6.7 | 0.30 | 0.34 | 0.36 | 39% | 91% | 144 |
|  |  | Full | SMOTE | 0.5 | 73% | 0.74 | 5.5 | 0.37 | 0.37 | 0.37 | 55% | 82% | 178 |
| Instance-Based | | |  |  |  |  |  |  |  |  |  |  |  |
|  | K-Nearest Neighbor (kKNN) | | |  |  |  |  |  |  |  |  |  |  |
|  |  | Full | None | 0.5 | 73% | 0.68 | 5.2 | 0.27 | 0.30 | 0.33 | 38% | 90% | 110 |
|  |  | Full | Over | 0.5 | 73% | 0.69 | 5.1 | 0.28 | 0.31 | 0.33 | 39% | 89% | 122 |
|  |  | Full | SMOTE | 0.6 | 75% | 0.67 | 6.6 | 0.27 | 0.31 | 0.34 | 34% | 93% | 109 |
|  | Weighted K-Nearest Neighbor (wKNN) | | | |  |  |  |  |  |  |  |  |  |
|  |  | Full | None | 0.5 | 73% | 0.69 | 5.1 | 0.31 | 0.33 | 0.34 | 45% | 86% | 145 |
|  |  | Full | Over | 0.5 | 74% | 0.69 | 5.5 | 0.33 | 0.35 | 0.35 | 46% | 86% | 161 |
|  |  | Full | SMOTE | 0.5 | 73% | 0.68 | 5.2 | 0.32 | 0.34 | 0.34 | 46% | 86% | 151 |
| Penalized Regression | | | | |  |  |  |  |  |  |  |  |  |
|  | Lasso | | |  |  |  |  |  |  |  |  |  |  |
|  |  | Full | None | 0.3 | 74% | 0.74 | 5.7 | 0.28 | 0.31 | 0.33 | 38% | 90% | 133 |
|  |  | Full | Over | 0.3 | 72% | 0.74 | 4.6 | 0.31 | 0.33 | 0.32 | 48% | 83% | 157 |
|  |  | Full | SMOTE | 0.3 | 72% | 0.74 | 4.6 | 0.31 | 0.33 | 0.32 | 48% | 83% | 157 |
|  | Ridge | | |  |  |  |  |  |  |  |  |  |  |
|  |  | Full | None | 1.0 | 69% | 0.68 | - | 0.00 | 0.00 | - | 0% | 100% | 110 |
|  |  | Full | Over | 0.3 | 73% | 0.69 | 4.8 | 0.28 | 0.31 | 0.32 | 41% | 87% | 122 |
|  |  | Full | SMOTE | 0.3 | 73% | 0.68 | 5.1 | 0.28 | 0.31 | 0.33 | 39% | 89% | 120 |
|  |  | MqTurb | None | 0.5 | 71% | 0.56 | 3.8 | 0.21 | 0.24 | 0.25 | 32% | 89% | 54 |
|  |  | MqTurb | Over | 1.0 | 69% | 0.63 | - | 0.00 | 0.00 | - | 0% | 100% | 96 |
|  |  | MqTurb | SMOTE | 0.3 | 72% | 0.64 | 4.8 | 0.33 | 0.34 | 0.34 | 52% | 82% | 141 |
|  |  | MPq | None | 0.4 | 70% | 0.63 | 3.7 | 0.28 | 0.29 | 0.29 | 48% | 80% | 102 |
|  |  | MPq | Over | 0.4 | 71% | 0.64 | 3.9 | 0.29 | 0.30 | 0.30 | 48% | 81% | 110 |
|  |  | MPq | SMOTE | 1.0 | 69% | 0.50 | - | 0.00 | 0.00 | - | 0% | 100% | 67 |
|  |  | MPqW | None | 0.3 | 70% | 0.64 | 3.5 | 0.26 | 0.27 | 0.27 | 46% | 80% | 99 |
|  |  | MPqW | Over | 0.3 | 63% | 0.65 | 3.0 | 0.27 | 0.24 | 0.24 | 64% | 62% | 108 |
|  |  | MPqW | SMOTE | 0.3 | 70% | 0.65 | 3.7 | 0.27 | 0.28 | 0.28 | 46% | 81% | 104 |
|  |  | MqW | None | 0.4 | 64% | 0.59 | 2.5 | 0.21 | 0.20 | 0.20 | 52% | 70% | 61 |
|  |  | MqW | Over | 0.4 | 67% | 0.60 | 2.6 | 0.19 | 0.20 | 0.20 | 39% | 80% | 59 |
|  |  | MqW | SMOTE | 0.3 | 73% | 0.62 | 4.8 | 0.28 | 0.31 | 0.32 | 41% | 87% | 98 |
|  |  | Pq | None | 0.4 | 71% | 0.63 | 3.8 | 0.21 | 0.24 | 0.25 | 32% | 89% | 70 |
|  |  | Pq | Over | 0.3 | 63% | 0.65 | 2.8 | 0.25 | 0.22 | 0.23 | 61% | 64% | 97 |
|  |  | Pq | SMOTE | 1.0 | 69% | 0.50 | - | 0.00 | 0.00 | - | 0% | 100% | 67 |
|  |  | PqW | None | 0.4 | 70% | 0.64 | 3.6 | 0.27 | 0.28 | 0.27 | 48% | 79% | 102 |
|  |  | PqW | Over | 0.4 | 71% | 0.65 | 3.8 | 0.22 | 0.25 | 0.26 | 34% | 88% | 78 |
|  |  | PqW | SMOTE | 0.3 | 72% | 0.65 | 4.2 | 0.29 | 0.30 | 0.31 | 45% | 84% | 108 |
|  |  | S | None | 0.3 | 57% | 0.68 | 3.5 | 0.27 | 0.21 | 0.25 | 80% | 46% | 119 |
|  |  | S | Over | 0.3 | 72% | 0.68 | 4.5 | 0.28 | 0.31 | 0.31 | 43% | 86% | 119 |
|  |  | S | SMOTE | 0.4 | 72% | 0.68 | 4.6 | 0.31 | 0.33 | 0.32 | 48% | 83% | 141 |
|  |  | W | None | 0.3 | 66% | 0.59 | 2.7 | 0.23 | 0.22 | 0.22 | 48% | 74% | 64 |
|  |  | W | Over | 1.0 | 69% | 0.50 | - | 0.00 | 0.00 | - | 0% | 100% | 71 |
|  |  | W | SMOTE | 0.3 | 66% | 0.60 | 2.8 | 0.24 | 0.23 | 0.23 | 50% | 74% | 76 |
| Rule-Based | | | |  |  |  |  |  |  |  |  |  |  |
|  | JRip | | |  |  |  |  |  |  |  |  |  |  |
|  |  | Full | None | 0.8 | 72% | 0.64 | 4.2 | 0.29 | 0.30 | 0.31 | 45% | 84% | 106 |
|  |  | Full | Over | 1.0 | 68% | 0.60 | 2.7 | 0.20 | 0.21 | 0.21 | 39% | 81% | 60 |
|  |  | Full | SMOTE | 0.8 | 74% | 0.65 | 5.7 | 0.28 | 0.31 | 0.33 | 38% | 90% | 106 |
|  | Partial Decision Trees (PART) | | | |  |  |  |  |  |  |  |  |  |
|  |  | Full | None | 0.1 | 57% | 0.60 | 2.2 | 0.19 | 0.16 | 0.17 | 64% | 54% | 67 |
|  |  | Full | Over | 0.2 | 57% | 0.60 | 2.2 | 0.19 | 0.16 | 0.17 | 64% | 54% | 67 |
|  |  | Full | SMOTE | 0.4 | 67% | 0.69 | 4.2 | 0.34 | 0.31 | 0.32 | 68% | 66% | 150 |
|  |  | Mq | None | 1.0 | 72% | 0.50 | - | 0.00 | 0.00 | - | 0% | 100% | 77 |
|  |  | Mq | Over | 1.0 | 72% | 0.50 | - | 0.00 | 0.00 | - | 0% | 100% | 74 |
|  |  | Mq | SMOTE | 1.0 | 70% | 0.50 | - | 0.00 | 0.00 | - | 0% | 100% | 70 |
|  |  | MqTurb | None | 0.2 | 72% | 0.64 | 4.4 | 0.31 | 0.32 | 0.31 | 48% | 82% | 120 |
|  |  | MqTurb | Over | 0.2 | 72% | 0.64 | 4.4 | 0.31 | 0.32 | 0.31 | 48% | 82% | 120 |
|  |  | MqTurb | SMOTE | 0.2 | 72% | 0.64 | 4.4 | 0.31 | 0.32 | 0.31 | 48% | 82% | 120 |
|  |  | MPq | None | 0.4 | 69% | 0.65 | 2.9 | 0.21 | 0.22 | 0.22 | 39% | 82% | 82 |
|  |  | MPq | Over | 0.4 | 69% | 0.65 | 2.9 | 0.21 | 0.22 | 0.22 | 39% | 82% | 82 |
|  |  | MPq | SMOTE | 0.2 | 59% | 0.61 | 2.5 | 0.22 | 0.19 | 0.20 | 66% | 56% | 77 |
|  |  | MPqW | None | 0.9 | 75% | 0.58 | 10.1 | 0.22 | 0.27 | 0.34 | 25% | 97% | 61 |
|  |  | MPqW | Over | 0.9 | 75% | 0.58 | 10.1 | 0.22 | 0.27 | 0.34 | 25% | 97% | 61 |
|  |  | MPqW | SMOTE | 0.8 | 67% | 0.60 | 2.5 | 0.18 | 0.19 | 0.20 | 38% | 81% | 55 |
|  |  | MqW | None | 0.7 | 66% | 0.54 | 1.9 | 0.12 | 0.13 | 0.13 | 30% | 82% | 30 |
|  |  | MqW | Over | 0.7 | 66% | 0.54 | 1.9 | 0.12 | 0.13 | 0.13 | 30% | 82% | 30 |
|  |  | MqW | SMOTE | 0.9 | 70% | 0.62 | 3.2 | 0.17 | 0.20 | 0.22 | 29% | 89% | 53 |
|  |  | Pq | None | 0.2 | 49% | 0.59 | 2.6 | 0.17 | 0.12 | 0.17 | 84% | 33% | 66 |
|  |  | Pq | Over | 0.2 | 49% | 0.59 | 2.6 | 0.17 | 0.12 | 0.17 | 84% | 33% | 66 |
|  |  | Pq | SMOTE | 0.2 | 60% | 0.60 | 2.2 | 0.19 | 0.17 | 0.18 | 59% | 60% | 65 |
|  |  | PqW | None | 0.9 | 75% | 0.58 | 10.1 | 0.22 | 0.27 | 0.34 | 25% | 97% | 61 |
|  |  | PqW | Over | 0.9 | 75% | 0.58 | 10.1 | 0.22 | 0.27 | 0.34 | 25% | 97% | 61 |
|  |  | PqW | SMOTE | 0.4 | 72% | 0.67 | 5.1 | 0.38 | 0.36 | 0.36 | 61% | 77% | 162 |
|  |  | S | None | 0.4 | 72% | 0.66 | 4.6 | 0.31 | 0.33 | 0.32 | 48% | 83% | 138 |
|  |  | S | Over | 0.4 | 72% | 0.66 | 4.6 | 0.31 | 0.33 | 0.32 | 48% | 83% | 138 |
|  |  | S | SMOTE | 0.5 | 72% | 0.68 | 4.6 | 0.31 | 0.33 | 0.32 | 48% | 83% | 143 |
|  |  | W | None | 0.1 | 52% | 0.59 | 3.3 | 0.22 | 0.17 | 0.22 | 84% | 38% | 85 |
|  |  | W | Over | 0.1 | 52% | 0.59 | 3.3 | 0.22 | 0.17 | 0.22 | 84% | 38% | 85 |
|  |  | W | SMOTE | 0.0 | 48% | 0.53 | 2.1 | 0.14 | 0.10 | 0.14 | 80% | 34% | 50 |
| Support Vector Machines (SVM) | | | | | | |  |  |  |  |  |  |  |
|  | Linear Hyperplane (SVM Linear) | | | | |  |  |  |  |  |  |  |  |
|  |  | Full | None | 0.5 | 73% | 0.71 | 5.2 | 0.32 | 0.34 | 0.34 | 46% | 86% | 161 |
|  |  | Full | Over | 0.6 | 75% | 0.71 | 6.0 | 0.32 | 0.35 | 0.36 | 43% | 89% | 157 |
|  |  | Full | SMOTE | 0.5 | 75% | 0.71 | 6.0 | 0.31 | 0.34 | 0.36 | 41% | 90% | 151 |
|  |  | Mq | None | 0.3 | 66% | 0.59 | 2.5 | 0.20 | 0.20 | 0.20 | 45% | 75% | 57 |
|  |  | Mq | Over | 0.3 | 66% | 0.59 | 2.5 | 0.20 | 0.20 | 0.20 | 45% | 75% | 57 |
|  |  | Mq | SMOTE | 1.0 | 69% | 0.59 | - | 0.00 | 0.00 | - | 0% | 100% | 84 |
|  |  | MqTurb | None | 1.0 | 69% | 0.44 | - | 0.00 | 0.00 | - | 0% | 100% | 72 |
|  |  | MqTurb | Over | 1.0 | 69% | 0.44 | - | 0.00 | 0.00 | - | 0% | 100% | 69 |
|  |  | MqTurb | SMOTE | 1.0 | 69% | 0.39 | - | 0.00 | 0.00 | - | 0% | 100% | 65 |
|  |  | MPq | None | 0.3 | 71% | 0.64 | 4.0 | 0.30 | 0.30 | 0.30 | 50% | 80% | 112 |
|  |  | MPq | Over | 0.3 | 71% | 0.64 | 4.0 | 0.30 | 0.30 | 0.30 | 50% | 80% | 112 |
|  |  | MPq | SMOTE | 0.4 | 72% | 0.66 | 4.3 | 0.30 | 0.31 | 0.31 | 46% | 83% | 124 |
|  |  | MPqW | None | 0.3 | 69% | 0.62 | 3.3 | 0.26 | 0.26 | 0.26 | 46% | 79% | 86 |
|  |  | MPqW | Over | 0.3 | 69% | 0.62 | 3.3 | 0.26 | 0.26 | 0.26 | 46% | 79% | 86 |
|  |  | MPqW | SMOTE | 0.4 | 66% | 0.64 | 3.3 | 0.29 | 0.27 | 0.27 | 59% | 70% | 107 |
|  |  | MqW | None | 0.3 | 57% | 0.56 | 2.1 | 0.18 | 0.15 | 0.16 | 62% | 55% | 51 |
|  |  | MqW | Over | 0.3 | 57% | 0.56 | 2.1 | 0.18 | 0.15 | 0.16 | 62% | 55% | 51 |
|  |  | MqW | SMOTE | 0.3 | 66% | 0.54 | 1.8 | 0.09 | 0.10 | 0.11 | 23% | 86% | 25 |
|  |  | Pq | None | 0.3 | 70% | 0.65 | 3.3 | 0.23 | 0.25 | 0.25 | 41% | 82% | 89 |
|  |  | Pq | Over | 0.3 | 70% | 0.65 | 3.3 | 0.23 | 0.25 | 0.25 | 41% | 82% | 89 |
|  |  | Pq | SMOTE | 0.3 | 70% | 0.66 | 3.5 | 0.25 | 0.27 | 0.26 | 43% | 82% | 102 |
|  |  | PqW | None | 0.4 | 70% | 0.62 | 3.0 | 0.19 | 0.21 | 0.21 | 32% | 86% | 58 |
|  |  | PqW | Over | 0.4 | 70% | 0.62 | 3.0 | 0.19 | 0.21 | 0.21 | 32% | 86% | 58 |
|  |  | PqW | SMOTE | 0.4 | 68% | 0.65 | 3.3 | 0.27 | 0.26 | 0.26 | 52% | 75% | 111 |
|  |  | S | None | 0.3 | 72% | 0.71 | 4.6 | 0.31 | 0.33 | 0.32 | 48% | 83% | 152 |
|  |  | S | Over | 0.3 | 72% | 0.71 | 4.6 | 0.31 | 0.33 | 0.32 | 48% | 83% | 152 |
|  |  | S | SMOTE | 0.3 | 72% | 0.71 | 4.6 | 0.31 | 0.33 | 0.32 | 48% | 83% | 152 |
|  |  | W | None | 0.4 | 66% | 0.54 | 1.9 | 0.12 | 0.13 | 0.13 | 30% | 82% | 29 |
|  |  | W | Over | 0.4 | 66% | 0.54 | 1.9 | 0.12 | 0.13 | 0.13 | 30% | 82% | 29 |
|  |  | W | SMOTE | 0.3 | 67% | 0.54 | 2.4 | 0.16 | 0.18 | 0.18 | 34% | 82% | 38 |
|  | Polynomial Hyperplane (SVM Polynomial) | | | |  |  |  |  |  |  |  |  |  |
|  |  | Full | None | 0.5 | 74% | 0.71 | 5.5 | 0.31 | 0.34 | 0.35 | 43% | 88% | 149 |
|  |  | Full | Over | 0.4 | 73% | 0.71 | 5.2 | 0.32 | 0.34 | 0.34 | 46% | 86% | 163 |
|  |  | Full | SMOTE | 0.3 | 67% | 0.68 | 4.3 | 0.35 | 0.31 | 0.32 | 68% | 67% | 152 |

^a^ To assess the relative information gain associated with using different feature types to build the models, two sets of analyses were performed. In the first set, each learner and the full set of features (Table S1) were used to develop full models. In the second set, the features listed in Table S1 were divided into four groups: Mq = microbial, Turb = turbidity, Pq= physicochemical water quality and air temperature collected on site; W= weather from publicly-available databases; S = spatial. Nested models were then built using different combinations of these feature types.

^b^ To assess the effect of resampling on model performance, two resampling methods [oversampling (over) and synthetic minority oversampling (SMOTE)] as well as no resampling (None) were used to develop three, separate models for each outcome-learner-feature type combination (e.g., nonpathogenic *Listeria* spp.-Ridge regressionFull Model).

^c^ After model tuning and training, predictive performance was assessed using the test data. The probability threshold was tuned to maximize kappa score, since the values of several performance measures (e.g., sensitivity) are dependent on this threshold.

^d^ ACC = Accuracy.

^e^ AUC = Area under the Curve. AUC equals the probability that the model will rank a randomly chosen positive sample higher than a randomly chosen negative sample. AUC ranges between 0 and 1, with AUC = 1.0 indicating perfect prediction. AUC ≤ 0.5 indicates that the model’s performance is equal to or worse than chance.

^f^ DOR = Diagnostic Odds Ratio. DOR ≤ 1 indicates a poor performing models (i.e., a model where a positive prediction is associated with a sample testing negative for nonpathogenic *Listeria* spp.). DOR > 1 indicates that a positive prediction is associated with the sample testing positive for nonpathogenic *Listeria* spp. [see (*2*)].

^g^ INF = Informedness, a measure of how informed the model is about both positive and negative samples. A value of 1 means that the pathogen status of all samples was correctly predicted, while a value of -1 implies the incorrect pathogen-status was predicted for all samples [see (*1*)].

^h^ Kappa score represents how much better the model performs compared to a model that randomly calls novel samples pathogen positive or negative. A score ≤ 0.0 indicates that the model is essentially useless, while a score = 1 indicates a model that always correctly identifies novel samples.

^i^ Matthew’s Correlation Coefficient. MCC ranges between -1 (the model always incorrectly predicts pathogen status) and 1 (the model always correctly predicts pathogen status). MCC ≤ 0.0 indicates that the model is equal to or worse than a model that randomly predicts pathogen status.

^j^ Sensitivity or true positive rate.

^k^ Specificity or true negative rate.

Figure S1: Log_10_ *E. coli* levels in training and test data samples that tested positive and negative for *L. monocytogenes* and nonpathogenic *Listeria* spp. (excluding *L. monocytogenes*). The colored lines represent the thresholds for agricultural water that were considered during development of the US Food Safety Modernization Act’s Produce Safety [126 MPN /100-mL (pink), 235 MPN /100-mL (blue) and 410 MPN /100-mL (green)].


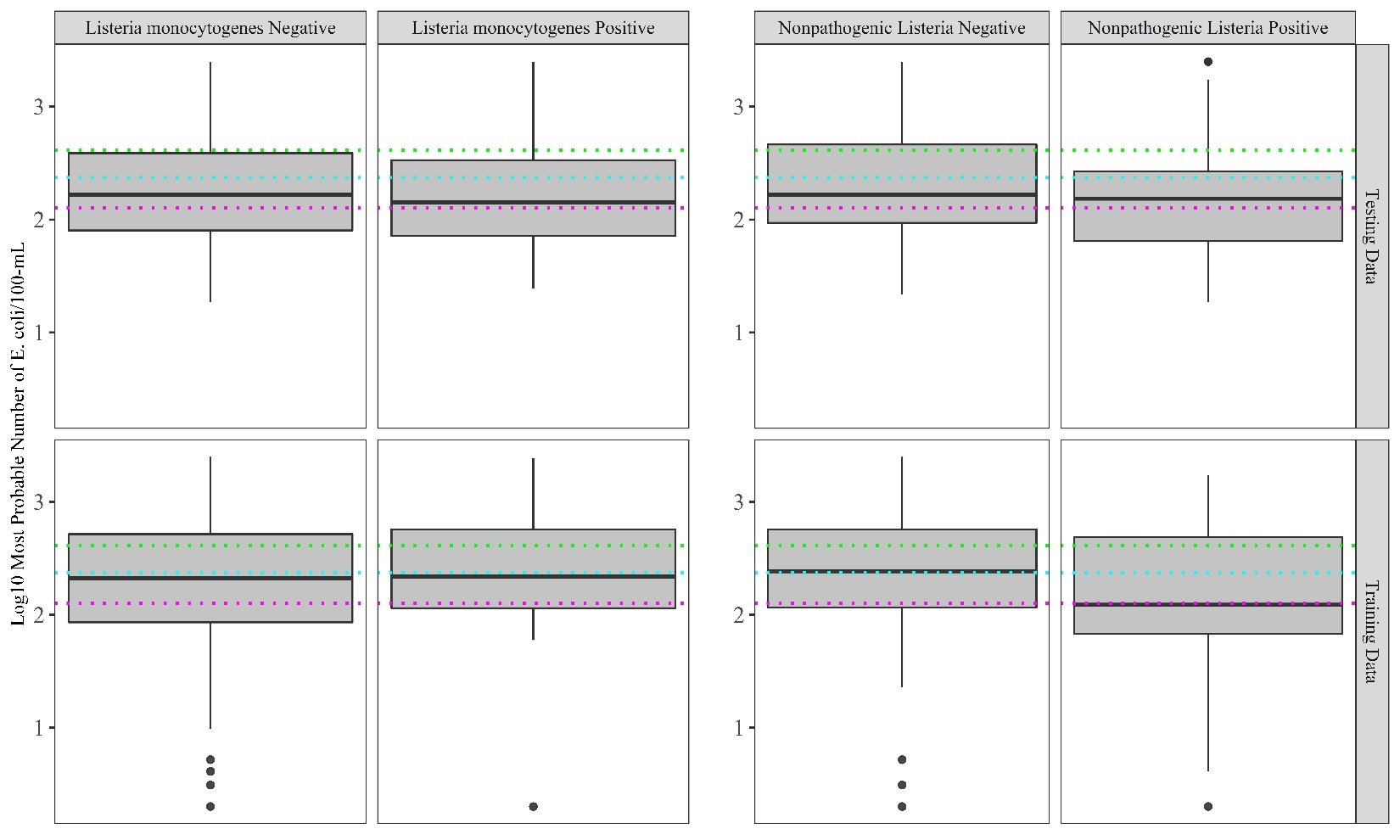


Figure S2: Area under the curve (AUC) and accuracy for models that predict *L. monocytogenes* presence in New York agricultural water. To facilitate readability models are faceted into full (left column) and nested (right column) models, and by resampling method [no resampling (top row), oversampling (middle row), and SMOTE (bottom row)]. The dotted red lines indicate the cut-offs for AUC (0.50) below which model’s performance is no better than chance.


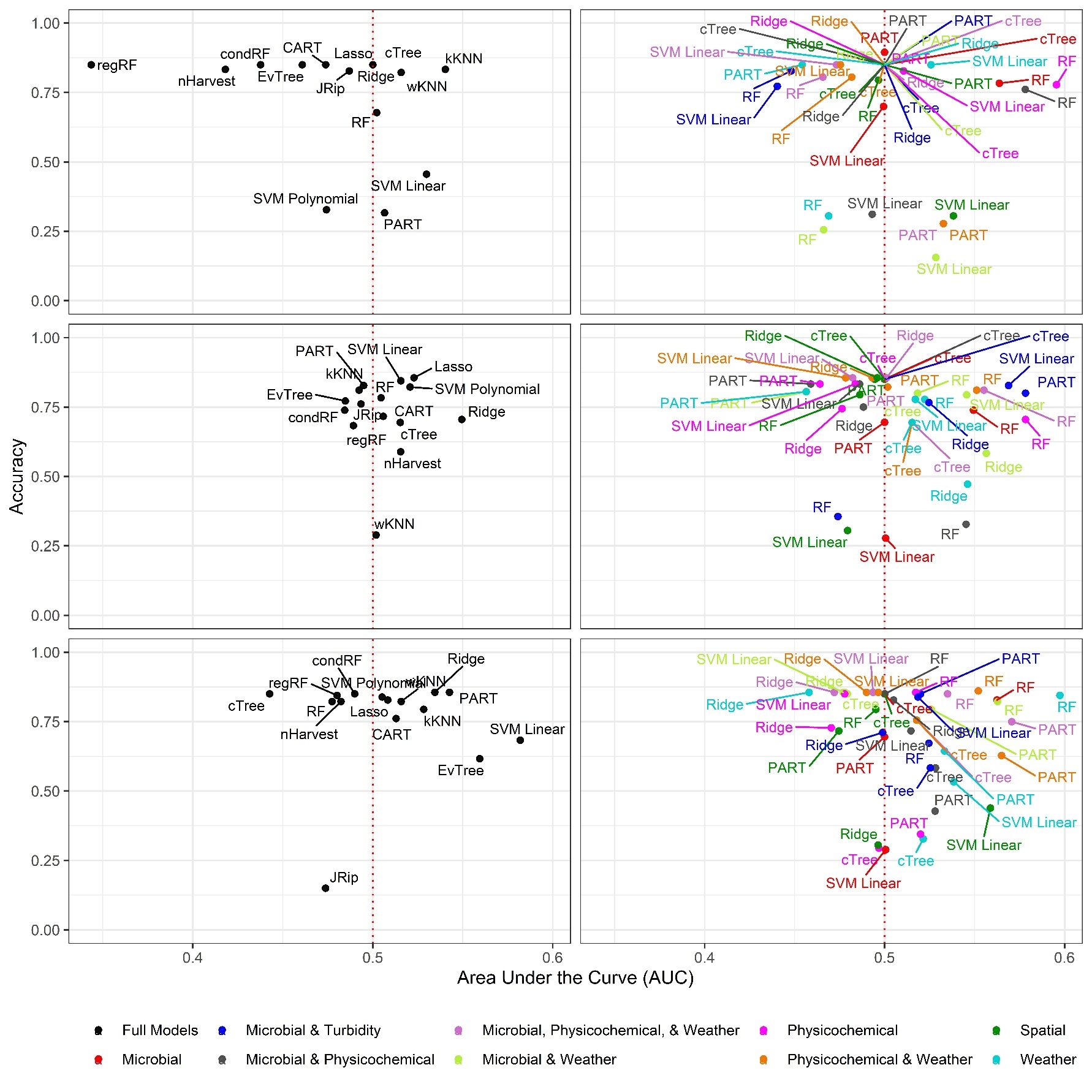


Figure S3: Area under the curve (AUC) and accuracy for models that predict nonpathogenic *Listeria* spp. (excluding *L. monocytogenes*) presence in New York agricultural water. To facilitate readability models are faceted into full (left column) and nested (right column) models, and by resampling method [no resampling (top row), oversampling (middle row), and SMOTE (bottom row)]. The dotted red lines indicate the cut-offs for AUC (0.50) below which model’s performance is no better than chance.


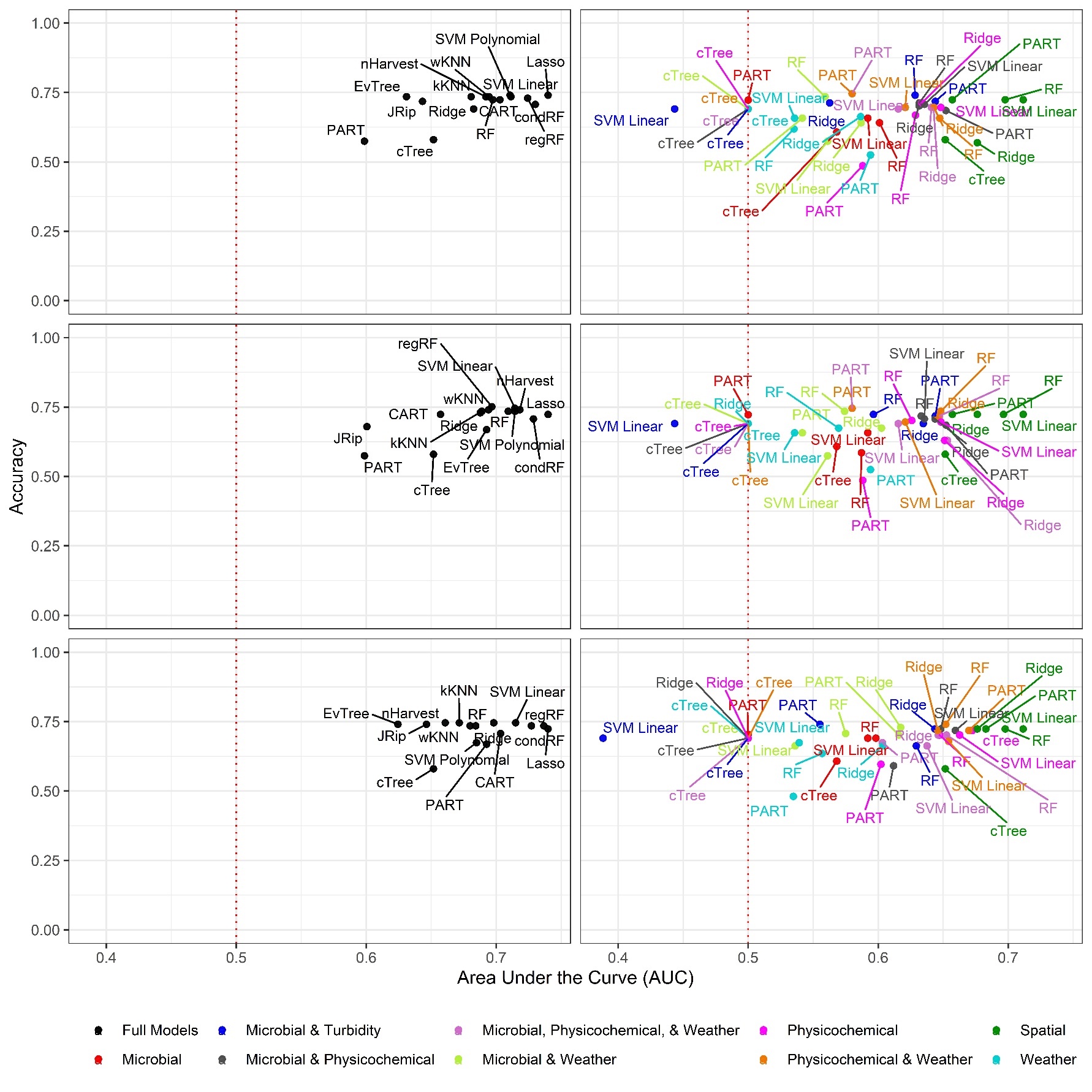


Figure S4: Diagnostic odds ratio (DOR) and Matthew’s Correlation Coefficient (MCC) for models that predict *L. monocytogenes* presence in New York agricultural water. To facilitate readability models are faceted into full (left column) and nested (right column) models, and by resampling method [no resampling (top row), oversampling (middle row), and SMOTE (bottom row)]. The dotted red lines indicate the cut-offs for DOR (1.0) and MCC (0.0) below which model’s performance is no better than chance.


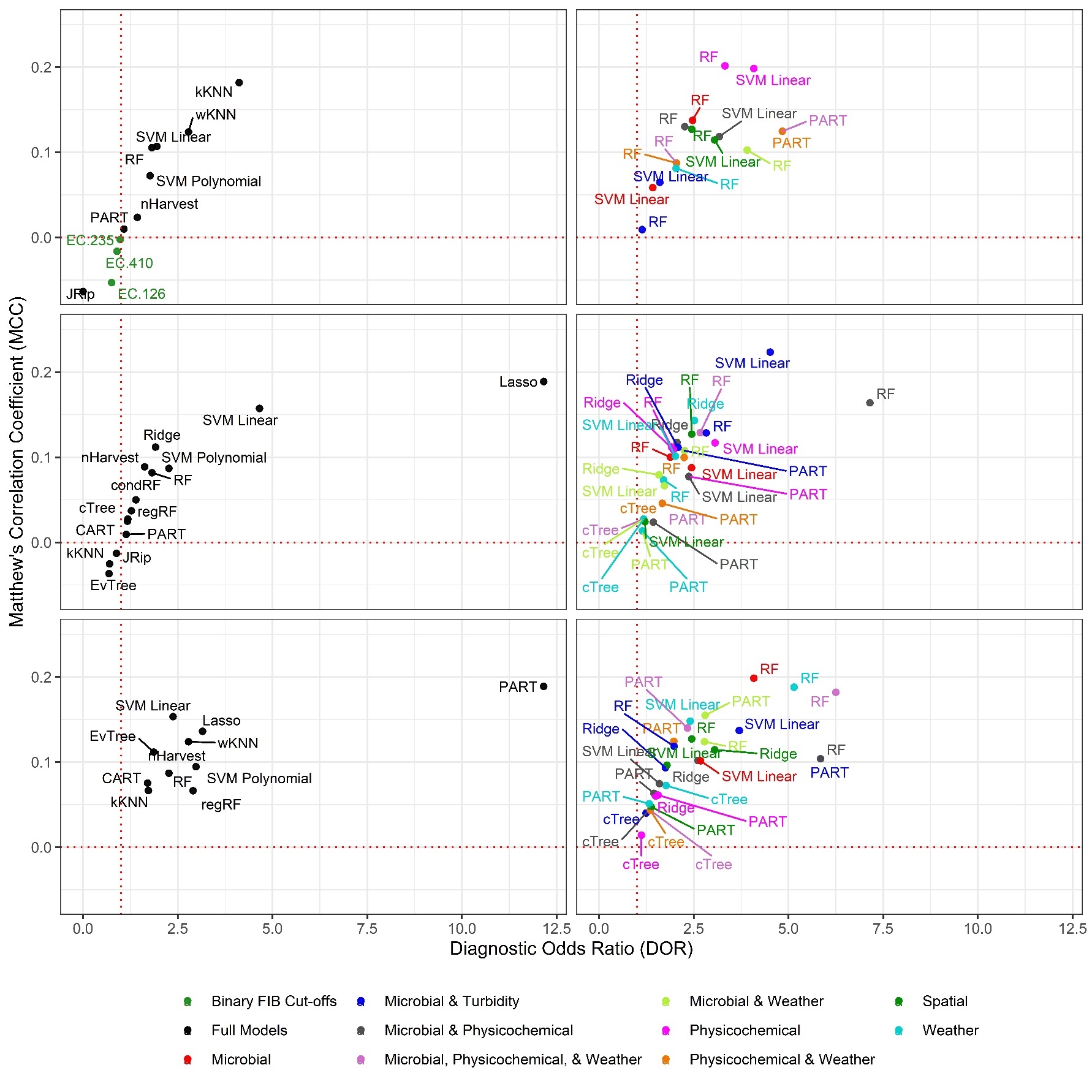


Figure S5: Diagnostic odds ratio (DOR) and Matthew’s Correlation Coefficient (MCC) for models that predict nonpathogenic *Listeria* spp. (excluding *L. monocytogenes*) presence in New York agricultural water. To facilitate readability models are faceted into full (left column) and nested (right column) models, and by resampling method [no resampling (top row), oversampling (middle row), and SMOTE (bottom row)]. The dotted red lines indicate the cut-offs for DOR (1.0) and MCC (0.0) below which model’s performance is no better than chance.


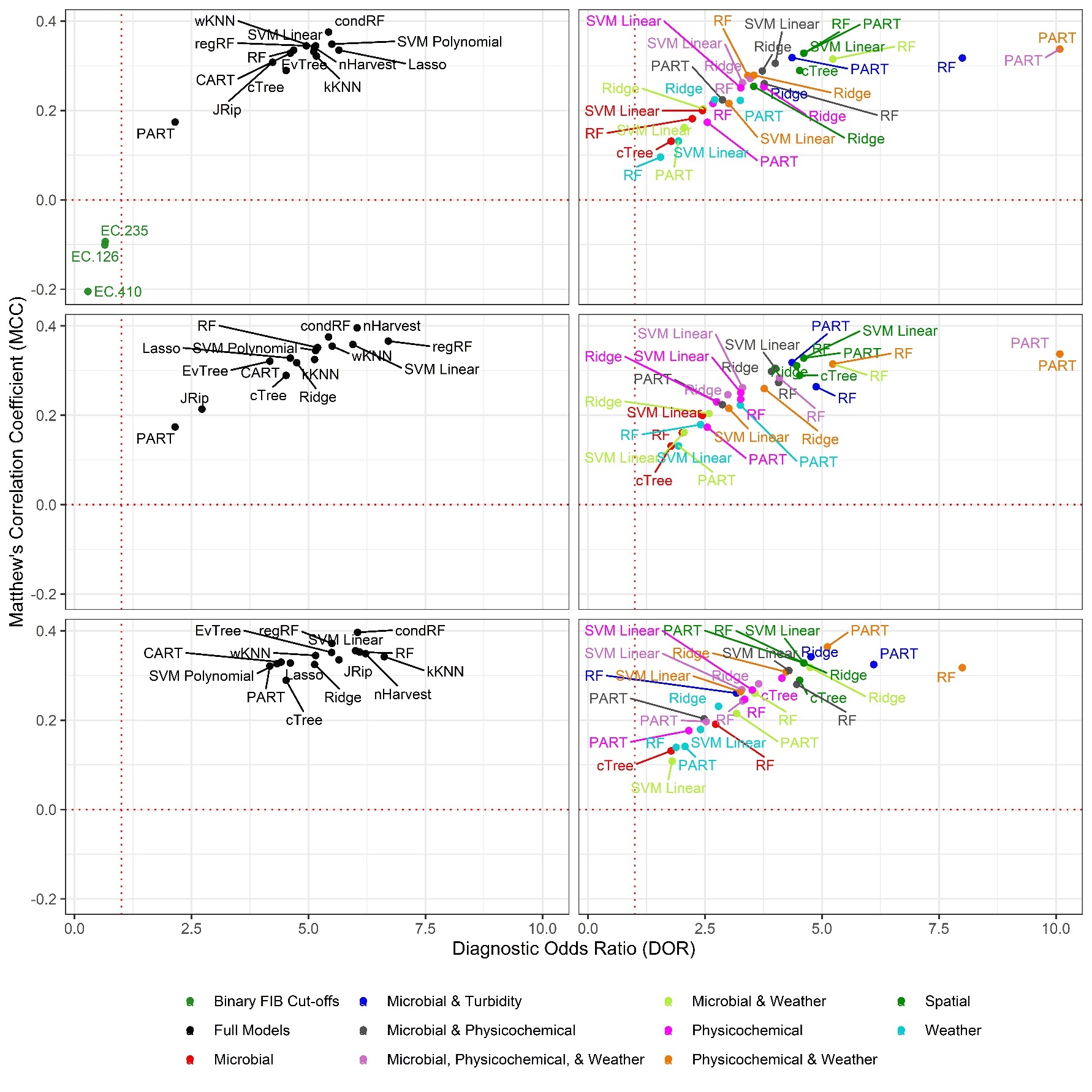


Figure S6: Convex hull graph showing model rank when predicting *L. monocytogenes* versus rank when predicting non-pathogenic *Listeria* spp. (excluding *L. monocytogenes*) presence for each combination of feature type and resampling approach. Convex hull graphs are useful for showing clustering by different types (in this case the feature types used when building each nested model). For example, note that spatial models consistently outperform models built using non-spatial features when the model aim is predicting non-pathogenic *Listeria* spp. (excluding *L. monocytogenes*) presence.

**
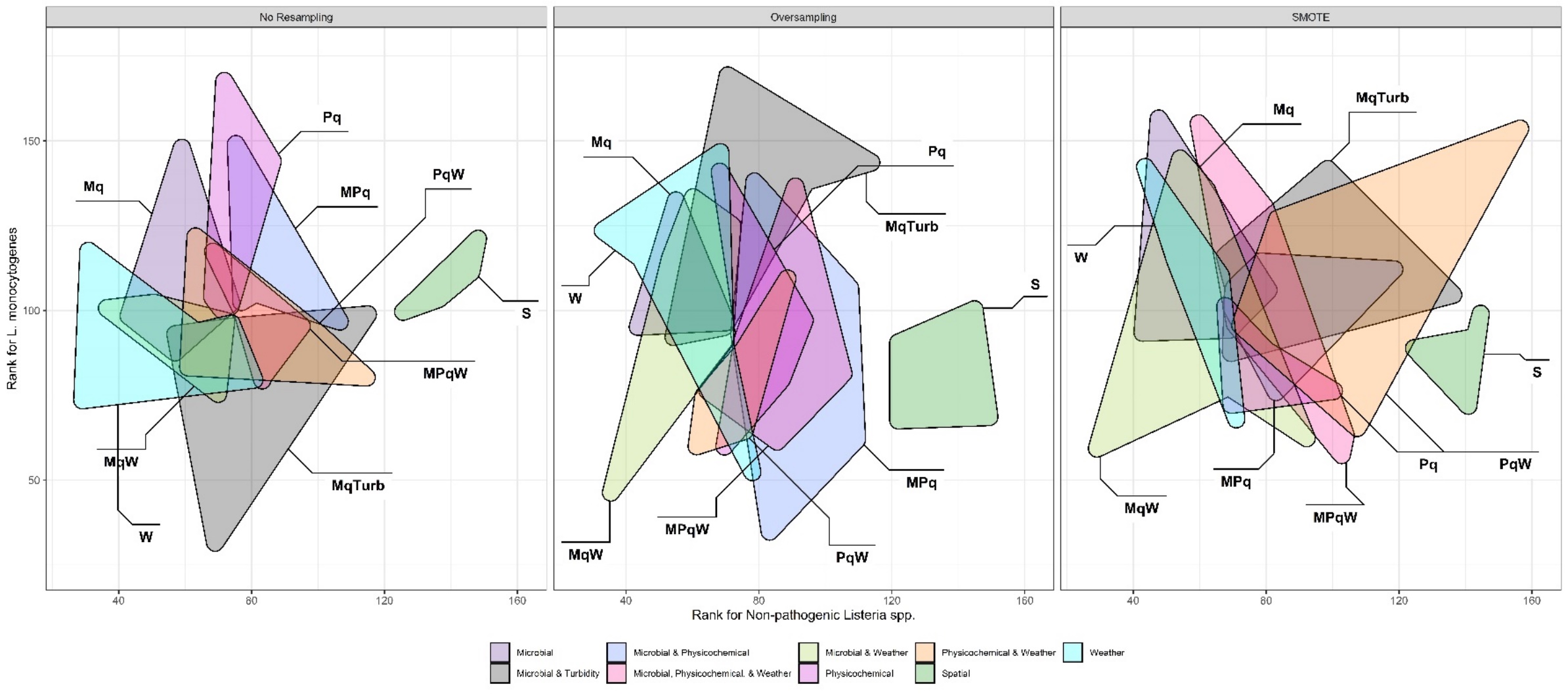
**

Figure S7: Plots showing the performance of the top-ranked full (A) and nested (B) *L. monocytogenes* models. The left column shows the top-ranked full (SVM linear, SMOTE) and nested (random forest, physicochemical, no resampling) models, while the right column shows the second-best performing full (evolutionary optimal trees, SMOTE) and nested (SVM linear, microbial & turbidity, oversampling) models. Within each set of graphs, the split quantiles plot (ranging in color from red to blue) shows how well the model is at accurately classifying positive and negative samples. The split quantiles plot is generated by sorting the test data from lowest to highest probability of *L. monocytogenes* detection based on the given model. The test data is then divided into quantiles (based on the percentile the probability falls into). The proportion of samples in each quantile that were actually *L. monocytogenes*-positive or negative were plotted. A good model would identify all low probability percentile samples (red) as negative (N) and all high probability percentile samples (blue) as positive (P). The density curve shows how well the model can distinguish samples that tested positive and negative for *L. monocytogenes*. The x-axis of is the probability of *L. monocytogenes* detection generated by the model, and the y-axis is density. The final plot is the receiver-operating curve (ROC) for the model; the x-axis is 1-Specificity and the y-axis is Sensitivity.


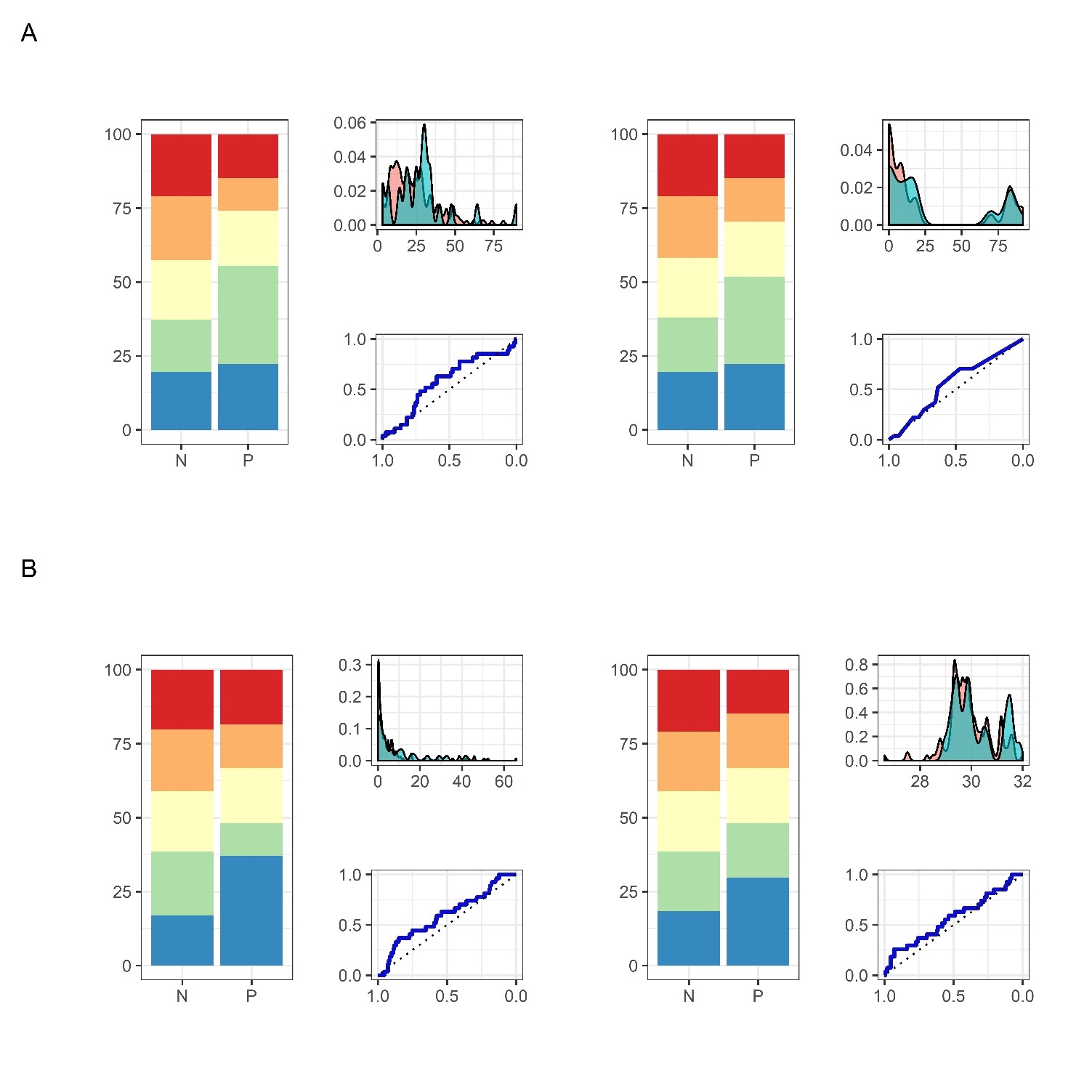

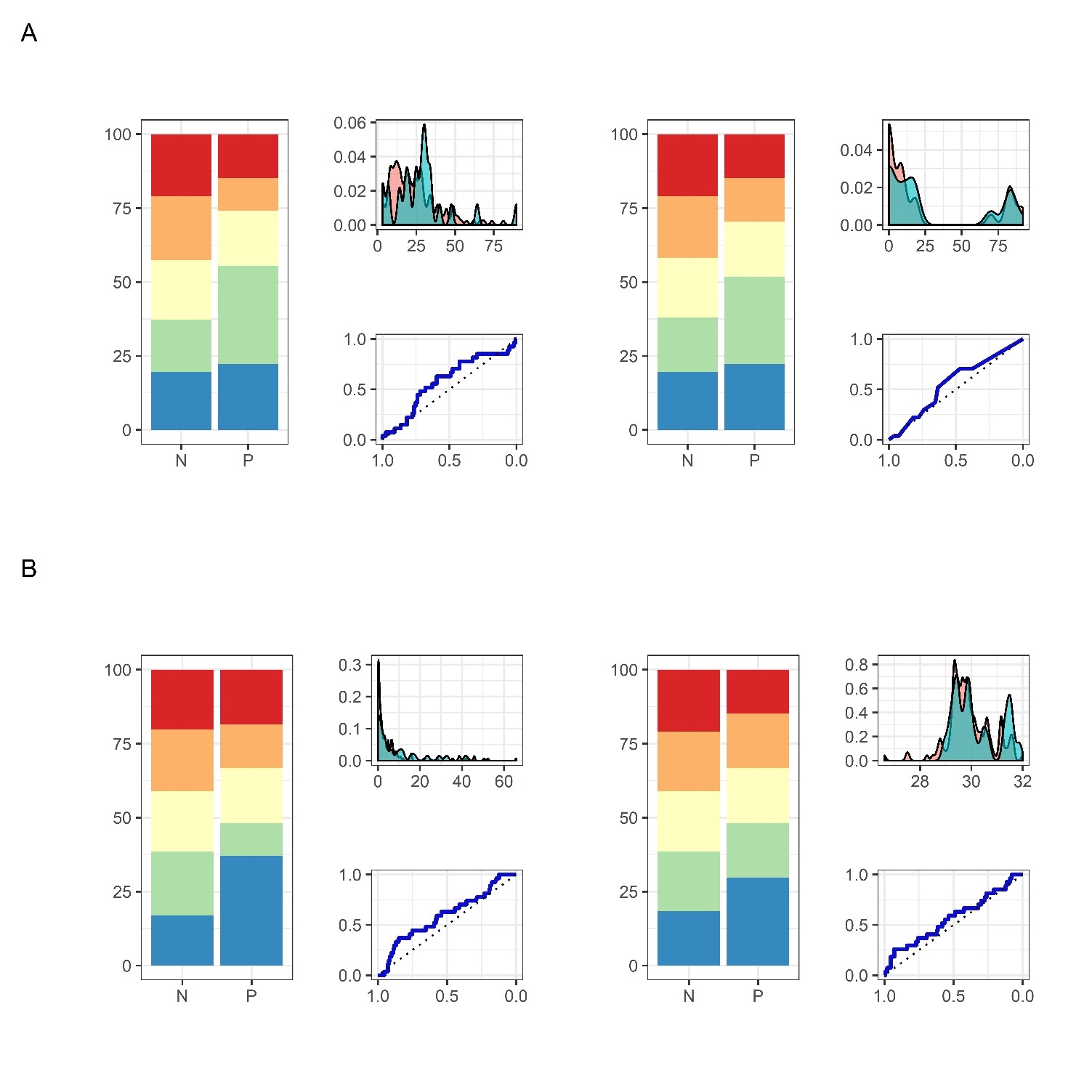


Figure S8: Plots showing the performance of the top-ranked full (A) and nested (B) nonpathogenic *Listeria* spp. (excluding *L. monocytogenes*) models. The left column shows the top-ranked full (conditional forest, SMOTE) and nested (partial decision trees, physicochemical & weather, SMOTE) models, while the right column shows the second-best performing full (conditional forest, no resampling) and nested (SVM linear, spatial, all resampling methods tied) models. Within each set of graphs, the split quantiles plot (ranging in color from red to blue) shows how well the model is at accurately classifying positive and negative samples. The split quantiles plot is generated by sorting the test data from lowest to highest probability of *Listeria* spp. detection based on the given model. The test data is then divided into quantiles (based on the percentile the probability falls into). The proportion of samples in each quantile that were actually *Listeria* spp. -positive or negative were plotted. A good model would identify all low probability percentile samples (red) as negative (N) and all high probability percentile samples (blue) as positive (P). The density curve shows how well the model can distinguish samples that tested positive and negative for *Listeria* spp. The x-axis of is the probability of *Listeria* spp. detection generated by the model, and the y-axis is density. The final plot is the receiver-operating curve (ROC) for the model; the x-axis is 1-Specificity and the y-axis is Sensitivity.


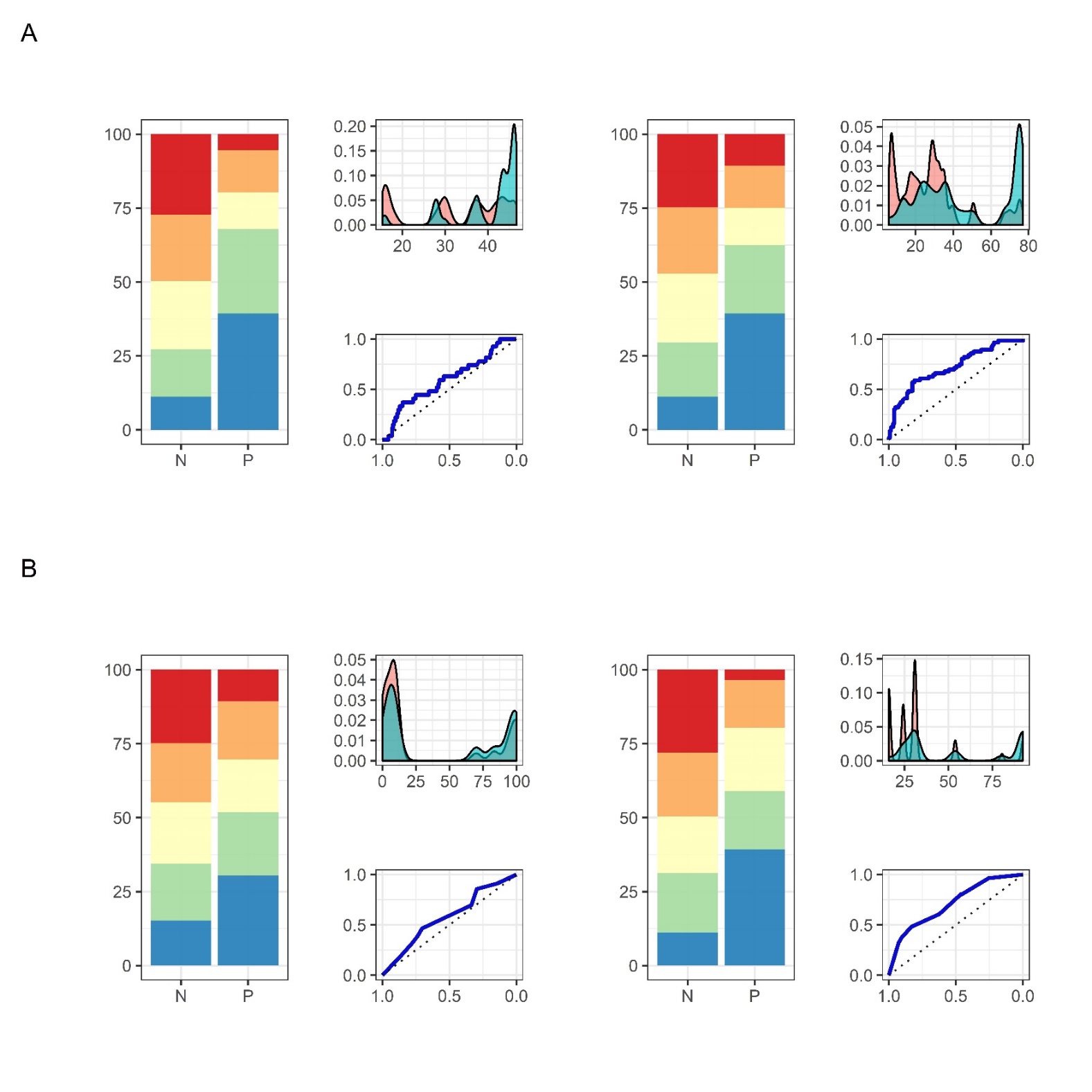


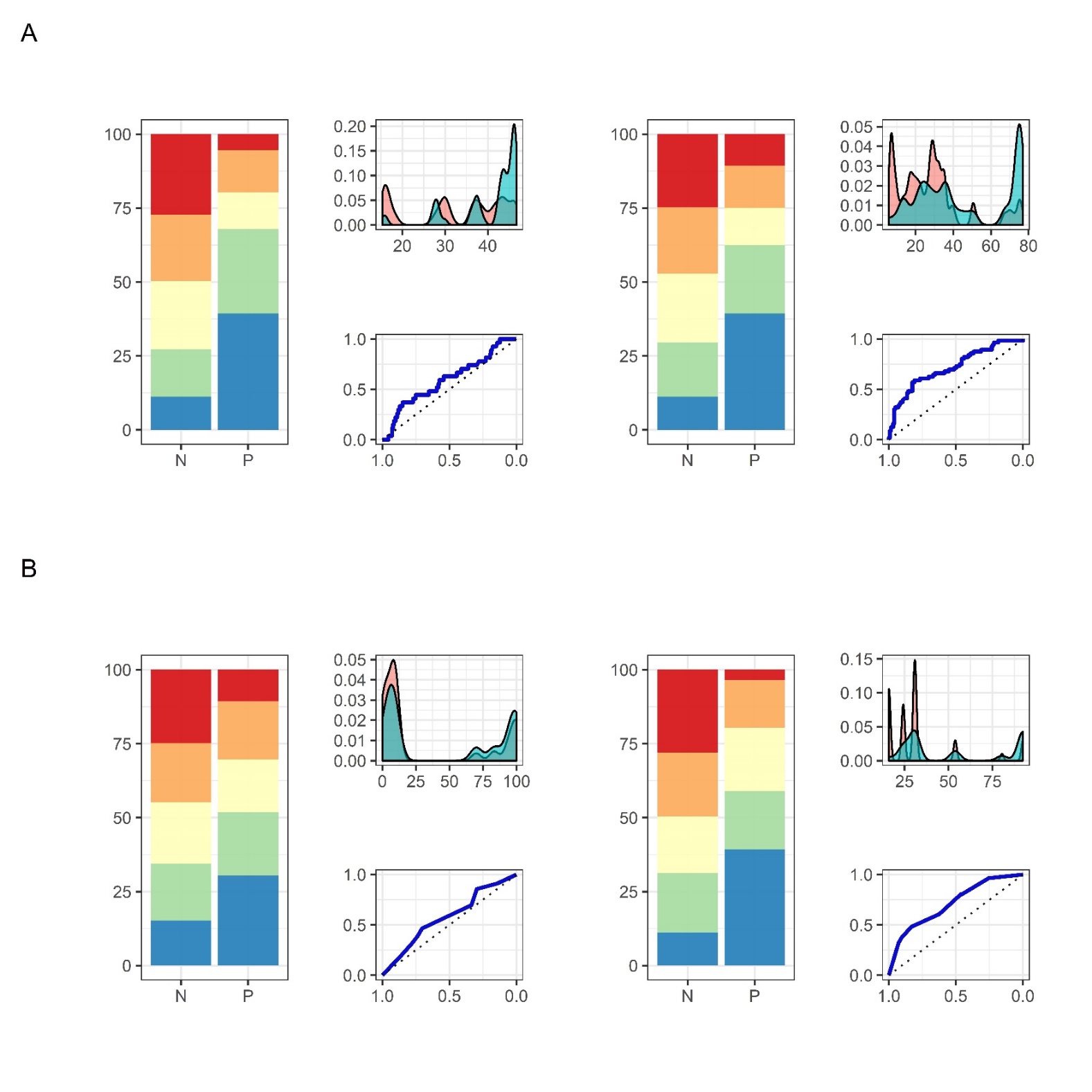
**References for Supplemental Materials:**

1. Powers, D. 2007. From Precision, Recall and F-Factor to ROC, Informedness, Markedness & Correlation. Australia.

2. Weller, D., N. Brassill, C. Rock, R. Ivanek, E. Mudrak, S. Roof, E. Ganda, and M. Wiedmann. 2020. Complex Interactions Between Weather, and Microbial and Physicochemical Water Quality Impact the Likelihood of Detecting Foodborne Pathogens in Agricultural Water. *Front. Microbiol.* Frontiers Media S.A. 11.

3. Anonymous 2006. Bridge Inventory Manual. Albany, NU.

4. Anonymous 2006. Culvert inventory and inspection manual.

5. Anonymous Dams.

6. Anonymous 2006. Guidance on outfall mapping: What is an outfall, and what should be mapped? Albany, NY.

7. Anonymous 2004. Methodology for the identification and survey of stormwater outfalls within designated MS4 locations for New York State DOT. Albany, NY.

8. Anonymous National Hydrography Database.

9. Anonymous 2019. NLCD 2016 Land Cover (CONUS). Sioux Falls, SD, SD.

10. Anonymous 2019. NLCD 2016 Percent Developed Imperviousness. Sioux Falls, SD, SD.

11. Anonymous NYS Large Culverts.

12. Anonymous Outfall and system mapping for illicit discharge detection and elimination (IDDE) in NY. Albany, NY, NY.

13. Anonymous Septic Systems, New York State, 2011 - CUGIR.

14. Anonymous Solid Waste Management Facilities | Open Data NY.

15. Anonymous State Pollutant Discharge Elimination System.

16. Anonymous 2004. Unified Stream Assessment: A User’s Manual. Ellicott City, MD.

17. Anonymous USGS National Transportation Dataset.
